# Supplementary material for: Relationship of CT densitometry to lung physiological parameters and health status in alpha-1 antitrypsin deficiency: initial report of a centralised database of the NIHR rare diseases translational research collaborative
Source: BMJ Open. 2020 Jun 30;10(6):e036045. doi: 10.1136/bmjopen-2019-036045 (PMC7328802; doi:10.1136/bmjopen-2019-036045)
Supplement: Supplementary data [file bmjopen-2019-036045supp001.pdf]

Lung Physiological equipment

Royal Free
integrated MasterScreen Pulmonary Function Testing machines (Carefusion, Germany) with the computer software JLab Lab Manager V5.32.0.
Cambridge
Jaeger Masterscreen
Royal Brompton
A CompactLab System (Jaeger, Hoechberg, Germany)
Nottingham
Spirometry - Spirometer NDD Easy On-PC
Plethysmography, DLCO - Jaeger Masterscreen PFT
Southampton
npire HDpft 4000 CAREstream Medical
Birmingham
Jaeger Masterscreen Pro lung function system (Jaeger Ltd, Hochberg, Germany)
Leicester
Medisoft HypeAir
