# Supplementary material for: Relationship of CT densitometry to lung physiological parameters and health status in alpha-1 antitrypsin deficiency: initial report of a centralised database of the NIHR rare diseases translational research collaborative
Source: BMJ Open. 2020 Jun 30;10(6):e036045. doi: 10.1136/bmjopen-2019-036045 (PMC7328802; doi:10.1136/bmjopen-2019-036045)

Supplementary table and figures

The relationship between CT densitometry and clinical measures in Patients with Alpha one Antitrypsin Deficiency: the NIHR Rare diseases Translational Research Collaboration.

Diana Crossley[1], James Stockley[2], Charlotte E Bolton[3], Nicholas S Hopkinson[4], Ravi Mahadeva[5], Michael Steiner[6], Tom Wilkinson[7], John R Hurst[8] ,Bibek Gooptu[6,9] and Robert A Stockley[2].

Supplementary table. Summary of correlation co-efficients between Quality of Life (QoL) measures and lung function parameters.

	CAT	SGRQ
**FEV_1** (L)	-0.41*	-0.51*
**FEV_1** (%predicted)	-0.41*	-0.55*
**FVC** (L)	-0.27*	-0.32*
**FEV_1/FVC** (%)	-0.26*	-0.38*
**Kco** (mmol/min/.kPa/.L)	-0.12	-0.11
**Kco** (% predicted)	-0.26*	-0.23*
**RV** (L)	0.35*	0.46*
**TLC** (L)	-0.07	0.02
**RV/TLC** (%)	0.30*	0.32*

(*=p<0.01)

Supplemental Figure S1. Scatter plots to show the relation between SGRQ and FEV_1 percent predicted. Data is shown for individual patient total SGRQ scores related to their FEV_1.

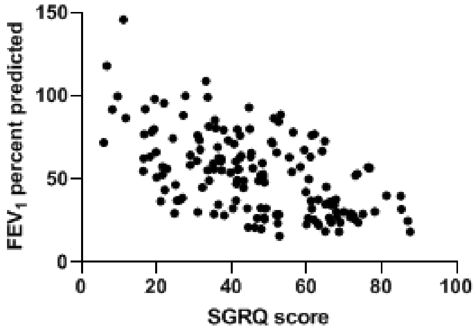

Supplemental figure S2. Scatter plots to show the relation between CAT and FEV_1 percent predicted. Data is shown for individual patient CAT scores related to their FEV_1.

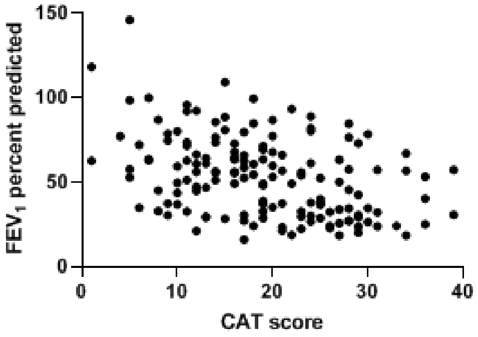

Supplement: Supplementary data [file bmjopen-2019-036045supp002.pdf]
