# Supplementary material for: Relationship of CT densitometry to lung physiological parameters and health status in alpha-1 antitrypsin deficiency: initial report of a centralised database of the NIHR rare diseases translational research collaborative
Source: BMJ Open. 2020 Jun 30;10(6):e036045. doi: 10.1136/bmjopen-2019-036045 (PMC7328802; doi:10.1136/bmjopen-2019-036045)
Supplement: Author's manuscript [file bmjopen-2019-036045.draft_revisions.pdf]

BMJ Open

BMJ Open is committed to open peer review. As part of this commitment we make the peer review history of every article we publish publicly available.

When an article is published we post the peer reviewers' comments and the authors' responses online. We also post the versions of the paper that were used during peer review. These are the versions that the peer review comments apply to.

The versions of the paper that follow are the versions that were submitted during the peer review process. They are not the versions of record or the final published versions. They should not be cited or distributed as the published version of this manuscript.

BMJ Open is an open access journal and the full, final, typeset and author-corrected version of record of the manuscript is available on our site with no access controls, subscription charges or pay-per-view fees (http://bmjopen.bmj.com).

If you have any questions on BMJ Open's open peer review process please email info.bmjopen@bmj.com

BMJ Open

The relationship between CT densitometry and clinical measures in Patients with Alpha one Antitrypsin Deficiency: the NIHR Rare diseases Translational Research Collaboration.

Journal:	*BMJ Open*
Manuscript ID	bmjopen-2019-036045
Article Type:	Original research
Date Submitted by the Author:	13-Dec-2019

Complete List of Authors:	Stockley, Robert; Queen Elizabeth Hospital<br>Crossley, Diana ; Queen Elizabeth Hospital<br>Stockley, James; Queen Elizabeth Hospital<br>Bolton, Charlotte; University of Nottingham,<br>Hopkinson, Nicholas; Royal Brompton Hospital, Respiratory Muscle Lab<br>Mahadeva, Ravi; Addenbrookes Hospital<br>Steiner, michael ; Institute for Lung Health, NIHR Leicester Biomedical Research Centre – Respiratory, University of Leicester, Glenfield Hospital,<br>Wilkinson, Tom; University of Southampton, Faculty of Medicine<br>Hurst, John; UCL Medical School,, Academic Unit of Respiratory Medicine<br>Gooptu, Bibek; Institute for Lung Health, NIHR Leicester Biomedical Research Centre – Respiratory, University of Leicester, Glenfield Hospital

Keywords:	Physiology < BASIC SCIENCES, GENERAL MEDICINE (see Internal Medicine), Thoracic medicine < INTERNAL MEDICINE, Computed tomography < RADIOLOGY & IMAGING, Emphysema < THORACIC MEDICINE, Chronic airways disease < THORACIC MEDICINE

SCHOLARONE™
Manuscripts

I, the Submitting Author has the right to grant and does grant on behalf of all authors of the Work (as defined in the below author licence), an exclusive licence and/or a non-exclusive licence for contributions from authors who are: i) UK Crown employees; ii) where BMJ has agreed a CC-BY licence shall apply, and/or iii) in accordance with the terms applicable for US Federal Government officers or employees acting as part of their official duties; on a worldwide, perpetual, irrevocable, royalty-free basis to BMJ Publishing Group Ltd ("BMJ") its licensees and where the relevant Journal is co-owned by BMJ to the co-owners of the Journal, to publish the Work in this journal and any other BMJ products and to exploit all rights, as set out in our licence.

The Submitting Author accepts and understands that any supply made under these terms is made by BMJ to the Submitting Author unless you are acting as an employee on behalf of your employer or a postgraduate student of an affiliated institution which is paying any applicable article publishing charge ("APC") for Open Access articles. Where the Submitting Author wishes to make the Work available on an Open Access basis (and intends to pay the relevant APC), the terms of reuse of such Open Access shall be governed by a Creative Commons licence – details of these licences and which Creative Commons licence will apply to this Work are set out in our licence referred to above.

Other than as permitted in any relevant BMJ Author's Self Archiving Policies, I confirm this Work has not been accepted for publication elsewhere, is not being considered for publication elsewhere and does not duplicate material already published. I confirm all authors consent to publication of this Work and authorise the granting of this licence.

For peer review only

The relationship between CT densitometry and clinical measures in Patients with Alpha one Antitrypsin Deficiency: the NIHR Rare diseases Translational Research Collaboration.

Diana Crossley[1], James Stockley[2], Charlotte E Bolton[3], Nicholas S Hopkinson[4], Ravi Mahadeva[5], Michael Steiner[6], Tom Wilkinson[7], John R Hurst[8] ,Bibek Gooptu[6,9] and Robert A Stockley[2].

[1]University of Birmingham, College of Medical and Dental Sciences, Institute of Inflammation and Ageing, Centre for Translational Inflammation Research, Queen Elizabeth Hospital, Mindelsohn Way, Birmingham, B15 2WB, United Kingdom.

[2]University Hospitals Birmingham NHS Foundation Trust, Queen Elizabeth Hospital Birmingham, Mindelsohn Way, Edgbaston, Birmingham, B15 2GW, United Kingdom.

[3]Department of Respiratory Medicine, NIHR Nottingham BRC respiratory theme, School of Medicine, The University of Nottingham, City Hospital Campus, Hucknall Road, Nottingham. NG5 1PB, United Kingdom.[4]National Heart and Lung Institute, Imperial College, Royal Brompton Hospital Campus, Fulham Road, London, SW3 6NP, United Kingdom.

[5]Department of Medicine, Cambridge NIHR BRC, University of Cambridge, Cambridge, CB2 0QQ, United Kingdom.

[6]Institute for Lung Health, NIHR Leicester Biomedical Research Centre – Respiratory, University of Leicester, Glenfield Hospital, Leicester, United Kingdom.

[7] Respiratory BRU, University of Southampton, University Road, Southampton, SO17 1BJ, United Kingdom.

[8] Respiratory, Royal Free Campus, London, NW3 2PF.

[9] King's College London, Guy's Hospital Site, Great Maze Pond, London, SE1 9RT.

Corresponding Author: Professor Robert A Stockley, University Hospitals Birmingham NHS Foundation Trust, Queen Elizabeth Hospital Birmingham, Mindelsohn Way, Edgbaston, Birmingham, B15 2GW, United Kingdom. E-mail: r.a.stockley@bham.ac.uk; Tel: +44 121 371 6808. ORCID 0000-0003-3726-1207

For peer review only

For peer review only - http://bmjopen.bmj.com/site/about/guidelines.xhtml

Abstract (word count 299)

Objectives

To establish a data base network for the study of alpha-1 antitrypsin deficiency (AATD) and compare the results to computed tomographic (CT) lung density as the most direct measure of emphysema.

Design

A central electronic data base was established to permit the upload of anonymised patient data from remote sites. Anonymised CT data was recorded onto disc and analysed at the coordinating centre and compared with the clinical features of the disease..

Setting

Tertiary referral centres with expertise in the management of AATD focussed on academic Biomedical Research units and Wellcome Clinical Research Facilities.

Participants.

Data was collected from 187 patients over 1 year from 8 UK academic sites. This included patient demographics, post bronchodilator physiology, health status and computed tomography. Analysis was undertaken at the coordinating centre in Birmingham.

Results.

Patient recruitment in the 12 months reached 94% of target (set at 200) covering the whole spectrum of the disease from those with normal lung function to very severe chronic obstructive lung disease. CT scan suitable for analysis was available from 147 (79%) of the patients. CT density analysed as the threshold for the lowest 15% of lung voxels, showed statistically significant relationships with the objective physiological parameters of lung function as determined by spirometric GOLD severity staging ($p< 0.001$) and carbon monoxide gas transfer ($p<0.01$). Density also correlated with subjective measure of quality of life ($p=0.02$).

Conclusions.

Establishment of the network for data collection and its transfer was highly successful facilitating future collaboration for the study of this rare disease and its management. CT densitometry correlated well with the objective clinical features of the disease supporting its role as the specific marker of the associated emphysema and its severity. Correlations with subjective measures of health, however, were generally weak indicating that other factors play a role.

For peer review only - http://bmjopen.bmj.com/site/about/guidelines.xhtml

Article summary

The strengths of the project has been the design and delivery of a centralised registry for alpha-1 antitrypsin deficiency enabling the whole spectrum of the disease to be characterised especially including those with more severe disease who are less able to travel long distances to single centres of excellence.

Recruitment and data collection was high.

The study provided further validation of the specificity of quantitative analysis of lung density for the assessment of emphysema in this “rare” disease.

The limitations of the study included the slight shortfall in patient recruitment over the 1 year target and slightly incomplete post bronchodilator physiological data entry.

A reduced number of CT scans were performed and available for analysis and although all patients filled in one of the health status tools this was not 100% for either of the individual tools.

Key Words.

Alpha-1antitrypsin deficiency, Emphysema, Computed Tomography lung physiology, Health status

For peer review only - http://bmjopen.bmj.com/site/about/guidelines.xhtml

INTRODUCTION

Alpha one antitrypsin deficiency (AATD) affects around 1 in 2500 individuals in the UK and increases the susceptibility to develop chronic obstructive pulmonary disease (COPD) with an early onset emphysema dominant phenotype and adult onset liver cirrhosis. AAT is the main plasma serine proteinase inhibitor and is predominantly made and secreted by the liver. It enters the tissues by simple diffusion especially in the presence of inflammation where it regulates the local activity of neutrophil proteinases (1). This is important in delicate tissue structures such as the alveolar region of the lung where uncontrolled serine proteinases can destroy connective tissues leading to alveolar destruction and the development of emphysema (2).

The study and treatment of rare diseases presents many problems including patient identification, referral to centres with relevant expertise, consistent demographic characterisation and importantly the design and delivery of appropriately powered research studies and clinical trials. With this in mind the UK National Institute for Health Research commissioned a series of projects in 2014 to develop networks for the study and deep phenotyping of rare diseases with the longer term aim of providing a consistent structure to facilitate collaboration between groups and with industrial partners to further research and therapeutic development.

In 1996 the ADAPT programme (Antitrypsin Deficiency and Programme for Treatment) was established in Birmingham as an investigator led, industry and research grants funded project in order to study AATD and its natural history. The programme depended on referrals, mainly from secondary care centres in the UK. Patients were invited to visit Birmingham on an annual basis for assessment as part of a clinical/ research programme (LREC 3359a). However, although successfully collating data on over 1000 deficient patients, those seen may be affected by bias due to referral patterns as well as to issues of geography and transport difficulties for annual visits. The latter issues may be especially relevant for the most severely affected patients. The ADAPT cohort may therefore underestimate the true impact of especially the severe end of the disease spectrum. In addition the evolution of a single centre impairs delivery of near patient care, clinical trials and iterative thought which can be handicapped by the absence of other local centres of excellence more convenient for patient attendance, management and especially, recruitment/participation in research and clinical trials.

For peer review only - http://bmjopen.bmj.com/site/about/guidelines.xhtml

The current article describes the AATD collaborative network and cross sectional patient demographics for a UK AATD cohort. In particular the relationship between lung densitometry as the most direct measure of emphysema and clinical demographic data is determined.

MATERIALS AND METHODS

Birmingham acted as the coordinating centre for the National Institute for Health Research Rare Diseases Translational Research Collaboration in AATD. Links were made with academic Biomedical Research Units where a specialist with an interest in AATD had been identified. The network had an initial target of 200 patients with AATD and the PiZZ genotype recruited across Birmingham, Nottingham, London Brompton, London UCL/Royal Free Hospital, Cambridge, Southampton and Leicester (see figure 1). The aim was to recruit 100 patients from Birmingham with mild and moderate disease and 100 patients with severe and very severe disease from the other centres such that the distance these patients had to travel was less than attending a single central facility. A standard protocol was provided to all centres based on that used in the core Birmingham centre and consisted of general demographic data required for in depth patient characterisation that would provide the basis of patient identification for inclusion in subsequent clinical trials using agreed standard operating procedures.

All patients gave informed consent and the study was approved for all sites by the local ethics committee (West Midlands – South Birmingham Research Ethics Committee, LREC 3359).

All subjects underwent full clinical examination, were scheduled to undergo full post bronchodilator lung function testing, determination of current health status using well established tools (St George's Respiratory Questionnaire; SGRQ and the COPD assessment tool; CAT). Where high resolution Computed Tomography had not been undertaken within the previous 2 years, a scan on full inspiration was taken using the following criteria:- subjects were scanned by spiral multi-slice CT of the chest in the supine position within 4 hours of administration of a short-acting bronchodilator. A low radiation dose (140kvp) was used, the slice thickness 5mm and increment 2.5mm, and a soft reconstruction algorithm (B30f) applied. The anonymised data was subsequently transferred to Birmingham on disc for quantitative analysis using Pulmo-CMS (Medis Medical Imaging, Leiden, Netherlands courtesy of Berend Stoel). Measurements for voxel index (% Voxels less dense than -950HU) and Perc15 (density threshold of the lowest 15% of the voxels) were assessed for the whole

For peer review only - http://bmjopen.bmj.com/site/about/guidelines.xhtml

lung such that greater Voxel index or lower Perc (PD)15 indicated a greater amount of emphysema.

Patient and Public Involvement.

The project was approved by 5 patients who completed a Traffic Light System (TLS) research proposal review form to give their opinion on the research project and the significance of its impact on clinical practice. A further patient representative was a formal member of the steering committee. The information will be part of future presentation to the patient group and summarised for dissemination in their annual newsletter once accepted for publication. Results will also be presented at international specialty meetings. This project represented a first step towards achieving the long term aims expressed by patients as part of the European lung Foundation survey of alpha-1 antitrypsin deficiency.

Statistical Analysis

Baseline characteristics are presented as median and interquartile range (IQR). The Pearson's or Spearman rank correlation coefficient between CT density and PFTs was calculated. Analysis of variance (ANOVA) or Kruskall-Wallis was used to assess differences between grouped data. Normality of the data was tested using the Shapiro Wilk test, and the level of significance was conventionally determined at $p<0.05$.

RESULTS

Baseline characteristics

There were 187 patients recruited to the study across the eight sites within the 1 year recruitment time. Confirmed post bronchodilator spirometry was available for 185 patients and carbon monoxide gas transfer for 181. Quantitative CT scan was obtained for 147 patients and 158 patients had completed both questionnaires analysed for measuring quality of life metrics (SGRQ and CAT).

For peer review only - http://bmjopen.bmj.com/site/about/guidelines.xhtml

The baseline characteristics are summarised in table 1. Data are presented as median and interquartile range to characterise the spread of patients studied. The median age was 60 years and the median post bronchodilator FEV_1 for the cohort was 51.1% predicted for age, sex, height and ethnicity (3). Of these 44 (24%) patients were graded as GOLD stage 4 at the time of study (4). 9 patients with AATD (5%) had an FEV1/FVC >0.7. The median CAT score for the whole group was 18.9 (12.0-25.0) and the median total SGRQ score was 45.2 (33.3-62.1).

Smoking history information was available for 178 patients. 79 were never smokers and all patients with a smoking history had ceased for more than 12 months at the time of recruitment. The median smoking history for the ex-smokers (n=99) was 17 pack years (IQR 8-27).

Cross sectional correlations

Lung function and CT densitometry

The relationship between CT density and lung function is shown in figure 2. There were significant correlations between both lung density parameters and the physiological parameters shown (p<0.001 all comparisons). Significant differences were also seen for CT density measurements as both the voxel index which quantifies the proportion of low density voxels below -950 HU and PD15 which quantifies the threshold below which 15% of the overall voxels are sited, between each of the GOLD severity stages (p<0.001) (figure 3). There was no correlation between RV/TLC and any of the densitometry parameters.

Table 1. Baseline Characteristics Table. Data is shown as median and interquartile ranges together with the number of patients where data was collected. All subjects were either never smokers or had ceased at least 1 year before the time of assessment. CAT score is only given for those where the total SGRQ score was also available.

Clinical Variable	Number of subjects	Median (IQR)
Age (yrs)	187	60.1 (50.9-66.9)
Sex	86M 101F (46%M, 54% F)	
Pack years for ex-smokers	99	17 (8-27)
FEV_1 (L)	185	1.4 (0.9-1.9)

For peer review only - http://bmjopen.bmj.com/site/about/guidelines.xhtml

FEV_1 percent predicted	185	51.1 (30.5-67.5)
FVC(L)	185	3.6 (2.9-4.8)
FVC percent predicted	185	105.7 (88.2-123.4)
FEV_1/FVC (%)	185	36.9 (28.5-49.8)
Kco percent predicted	181	56.3 (46.0 - 69.6)
RV/TLC (%)	140	41.5 (33.0-52.7)
Voxel index -950 HU (%)	147	24.5 (15.4-35.2)
Perc15 (HU)	147	-965.2 (-974.8—950.7)
Total CAT score	158	18.9 (12.0-25.0)
Total SGRQ score	158	45.2 (33.3-62.1)

For peer review only

Following adjustment of all these relationships for age, sex and pack years, the relationship between density as measured by PD15 and voxel index remained significant ($p<0.001$) with no evidence of collinearity (see table 2).

Table 2. Multivariate linear regression models between CT density and Lung function.

Data is shown for the regression analysis of both CT parameters and baseline lung function. "pp" is the percent predicted

CT density	Lung function parameter	Unstandardised co-efficient	Standard error
PD15	FEV_1pp	0.63	0.1 ($p<0.001$)
	FEV_1/FVC (%)	0.94	0.1 ($p<0.001$)
	Kcopp	0.55	0.1($p<0.001$)
Voxel index (%)	FEV_1pp	-0.48	0.1 ($p<0.001$)
	FEV_1/FVC (%)	-0.67	0.1 ($p<0.001$)
	Kcopp	-0.5	0.8 ($p<0.001$)

Quality of life measures and Lung Function

Within the cohort, the CAT test scores ranged between 1 and 39/40 (median=18.9; IQR 12-25) and related to disease severity as defined by the GOLD stage and summarised in figure4 ($p<0.0001$). The median SGRQ was 45.2 (33.3-62.1) and also related to the GOLD stage ($p<0.001$) shown in figure 4. ($p<0.001$). The median and IQR for CAT and SGRQ in each GOLD stage is summarised in table 3.

For peer review only - http://bmjopen.bmj.com/site/about/guidelines.xhtml

For peer review only

Table 3. Quality of Life data across each GOLD stage (4). The number of patients for each GOLD stage is shown together with the median and IQR data for the CAT and SGRQ total score.

GOLD Stage	Patient number	CAT median (IQR)	Patient number	SGRQ median (IQR)
FEV1/FVC>0.7	9	12 (6-21)	9	27.1 (9.7-48.0)
1	13	15 (9-21)	12	33.4 (19.3-35.7)
2	63	16 (12-21)	63	41.5 (30.9-55.4)
3	39	19 (11-25)	39	48.8 (36.5-67.3)
4	53	20 (13.5-27)	33	62.0 (36.5-67.3)

There were significant correlations (Supplemental Table 1) between both QoL measures and spirometry, as measured by FEV_1 (percent predicted), FVC (percent predicted) , FEV_1/FVC (%), and with gas transfer co-efficient, Kco (percent predicted) and gas trapping as measured by RV/TLC % ($p<0.01$ all comparisons). Supplemental figures 1 and 2 summarise the correlations for SGRQ and CAT with FEV_1 (% predicted)

Quality of life measures and CT density

Both Quality of life measures were worse the greater the emphysema as quantified by the lung density. There was a significant relationship between voxel index and CAT ($r= 0.18$, $p=0.019$) and a weaker relationship with PD15 ($r=0.15$, $p=0.043$) (figure 5). Total SGRQ correlated significantly with both measures of CT density, although again the relationship was weak ($r^2<0.1$) (see figure 5).

Discussion

The current paper reports demographic data collated from a unique NIHR funded project to establish collaborative cohorts of patients with rare diseases focussed on academic Biomedical Research units and Wellcome Clinical Research Facilities. The main purpose of this project was to recruit 200 patients with AATD (PiZZ genotype) to a central data base over a 1 year period as a basis for ongoing collaborative research. The aim of the

For peer review only - http://bmjopen.bmj.com/site/about/guidelines.xhtml

collaborative was to recruit and characterise patients with AATD in depth across centres with a wide spectrum of severity . Importantly we wished to include those with very severe disease (GOLD stage 4) as quantified by baseline FEV_1 (4) in centres local to their dwelling who may not wish to travel to a more central facility. Our data confirmed the feasibility of this approach and importantly the data produced similar values and demographic relationships to those seen historically in the single centre ADAPT programme in Birmingham.

We have confirmed the feasibility of this approach with recruitment from multiple academic centres in this limited time period reaching 94% of the target. Of these 98% had confirmed post bronchodilator spirometry , 92% had gas transfer measurements and 85% had both SGRQ and CAT measurement of health status although most patients had CAT recorded. CT scans were performed at baseline using a fixed protocol and copied anonymously on to disc for analysis in Birmingham. This resulted in a total of 147 scans available for analysis (79% of patients) enabling the relationships to clinical parameters to be explored.

The patients showed a wide spectrum of disease including 33 classified as GOLD stage 4. Health status showed a relationship to spirometric severity with worsening for each stage although (as expected) with a wide range and major group overlap as reported from the larger ADAPT data base(5) and other studies of non AATD COPD (6). Importantly the patients in GOLD stage 4 had a median total SGRQ score 62.0 (IQR 36.5-67.3) which was similar to that for 51 stage 4 patients in the ADAPT data base (extracted from ref 5) with a median value of 59.1 (IQR 51.8-72.0). This suggests that our previous data was not influenced by acquisition bias as a result of travel to a single centre being influenced by distance and/or patient health. In addition it indicates that GOLD 4 patients can be readily recruited from multiple sites for research, audit and clinical trials purposes.

Relationship of lung densitometry (the most direct measure of pathological emphysema) to objective parameters of lung function was similar to that seen in other studies (7,8) but better for gas transfer than spirometry (consistent with the former being a more direct measure of alveolar dysfunction as seen in emphysema) and certainly better than the weak relationship to air trapping (RV/TLC) and the subjective health status measures. COPD even in AATD is a

For peer review only - http://bmjopen.bmj.com/site/about/guidelines.xhtml

multifaceted condition although mainly characterised by the presence of panacinar emphysema localised to the lung bases which is the dominant pathological phenotype. Lung densitometry is the most direct measure of this process as demonstrated pathologically (9) and its severity. All other measures are indirect surrogate markers of this process and although densitometry in AATD relates to exercise capacity (10) spirometry and gas transfer (11) and health status (12) it remains the best predictor of mortality in this disease (13). In addition it is the most sensitive measure of progression in AATD (14) and hence its inclusion as the primary outcome in clinical trials which enables such studies to be sufficiently powered in such a rare disease.

The study was successful in enabling collaboration between academic centres by providing recruitment and in depth phenotyping of a group of patients with AATD and a wide range of physiological impairment to an agreed standard. All subjects also had plasma taken and stored for the development and validation of assays to monitor serine proteinase footprints (15) and other disease specific biomarkers. In addition the network provides baseline cohort data for long term follow up and importantly a well characterised patient data base for collaboration with industry (one of the key aims for the NIHR programme). Furthermore it conforms to the key elements outlined by the European Respiratory Society strategy document for the management of AATD (16); namely establishment of registries, comprehensive assessment and review by specialists with an interest in AATD. It also provides the background for linking with the European Union rare diseases collaborative and the European Alpha-1 Research Collaboration (EARCO) (17).

Nevertheless the study has some limitations. Many of the patients had undergone CT scanning as part of their clinical assessment prior to the establishment of the collaborative and hence it was not deemed ethical to repeat the procedure in the short term for densitometry analysis as part of a phenotyping study. However the parameters are set for all future scans in this patient population both at baseline and for monitoring progression. The documentation of health status was not perfectly consistent although either the SGRQ or CAT was performed in all subjects. Only 85% had the SGRQ documented whereas 95% had the CAT documented which may reflect the latter's ease of administration and practical clinical utility (4). Our analysis reported here only included those patients in whom both had been measured.

For peer review only - http://bmjopen.bmj.com/site/about/guidelines.xhtml

Currently only the SGRQ has been accepted as a validated tool for patient reported outcomes in clinical trials (18) whereas the CAT has become an accepted measure in patient management as supported by GOLD(4) and is often also reported in clinical studies and trials.

In summary the project successfully recruited to 94% of target in the one year time frame and the majority underwent deep phenotyping to a set standard for future patient care and assessment consistent with the view of the European Respiratory Society strategy document for AATD (16). The collaboration and expertise of participants provides a firm structure for future management of AATD in the UK. Importantly it provides validation that previous patient assessment as part of the ADAPT programme reflects patients across the spectrum of disease severity and not a patient acquisition bias related to distance or travel. Furthermore it demonstrates an intercalated network that can harmonise activity and research outcomes using standard methodologies. These points give reassurance that patient monitoring and care can be provided at multiple sites to the same standard, factors that are both critical in patient convenience and reassurance, as expressed by patients in the recent ERS AATD strategy document (16). Importantly it provides validation of CT densitometry for characterisation of the pathological nature and severity of the disease and places this in perspective with other measures routinely used for monitoring. Because of its specificity it should become an essential part of AATD assessment (16). Finally it provides an essential readymade resource for the delivery of research, clinical studies and trials especially for the pharmaceutical industry and a UK resource for patient/specialist research collaboration.

For peer review only - http://bmjopen.bmj.com/site/about/guidelines.xhtml

Author Contributions

The project concept was developed by RAS. JAS, CEB, NSH, JRH, BG, RM, MS, TAW and RAS established the network as part of the NIHR rare diseases call, collated clinical, physiological and radiological data across each contributing organisation. DC analysed the quantitative CT scans and the related data. DC drafted and refined the manuscript with RAS. All authors reviewed, modified and approved the final manuscript. None of the authors have financial relationships with any organisations in the previous three years that might have an interest in the submitted work; no other relationships or activities that could appear to have influenced the submitted work. However, RAS sits on several advisory boards for pharmaceutical companies with or developing treatments for alpha-one antitrypsin deficiency and NSH is currently recruiting to a phase 2 study in these patients sponsored by Mereo Biopharma.

Access to data

Anonymised data is stored on the central data base in Birmingham and site specifically at the collaborative sites. Access to data can be obtained by approval of the registry holders following application to the senior author

Acknowledgements: We would like to thank Anita Pye, the study coordinator for her assistance and support and Mr J Beggs the patient representative on the Steering committee.

The Research and Network was funded by The NIHR rare diseases TRC and coordinated from the National Institute for Health Research (NIHR)/Wellcome Trust Birmingham Clinical Research Facility,. *NIHR Nottingham BRC respiratory theme , Cambridge NIHR BRC ,NIHR Leicester BRC – respiratory theme, Respiratory BRU Southampton, NIHR Respiratory BRU at Royal Brompton and Harefield NHS Foundation Trust and Imperial College, London*

The views expressed are those of the authors(s) and not necessarily those of the NHS, the NIHR or the Department of Health.

For peer review only - http://bmjopen.bmj.com/site/about/guidelines.xhtml

References

1. Stockley RA.. The measurement of soluble proteins in lung secretions. Thorax. 1984; 39: 241-247.

2. Stockley RA. Alpha-1-antitrypsin and the pathogenesis of emphysema. Lung. 1987; 165: 61-77.

3. Quanjer PH, Tammeling GJ, Cotes JE, Pedersen OF, Peslin R, Yernault JC. Lung volumes and forced ventilatory flows. Eur Respir J. 1993;6 Suppl 16:5-40.

4. Vogelmeier CF, Criner GJ, Martinez FJ, Anzueto A, Barnes PJ, Bourbeau J, et al.A.Global Strategy for the Diagnosis, Management, and Prevention of Chronic Obstructive Lung Disease 2017 Report. GOLD Executive Summary. Am J Respir Crit Care Med. 2017 Mar 1;195(5):557-582

5. Stockley RA, Edgar RG, Starkey S, Turner AM. Health status decline in α-1 antitrypsin deficiency: a feasible outcome for disease modifying therapies?
Respir Res. 2018; Jul 20;19(1):137.

6. Tabberer M, Benson VS, Gelhorn H, Wilson H, Karlsson N, Müllerova H, et al. The COPD Biomarkers Qualification Consortium Database: Baseline Characteristics of the St George's Respiratory Questionnaire Dataset. Chronic Obstr Pulm Dis. 2017 Mar 13;4(2):112-123

7. Kim SS, Seo JB, Lee HY, et al. Chronic obstructive pulmonary disease: lobe-based visual assessment of volumetric CT by using standard images-comparison with quantitative CT and pulmonary function test in the COPDGene study. Radiology. 2013;266(2):626–635

8. Barjaktarevic I, Springmeyer S, Gonzalez X, Sirokman W, Coxson HO, Cooper CB. Diffusing capacity for carbon monoxide correlates best with tissue volume from quantitative CT scanning analysis. Chest. 2015;147(6):1485–1493.

For peer review only - http://bmjopen.bmj.com/site/about/guidelines.xhtml

9. Gevenois PA, de Maertelaer V De Vuyst P, Zanen J and Yernault JC.Comparison of computed density and macroscopic morphometry in pulmonary emphysema. Am J Resp Crit Care Med. 1995;152:653-657.

10. Dowson LJ, Newal Cl, Guest PJ, Hill SL and Stockley RA. Exercise capacity predicts health status in α1-antitrypsin deficiency. Am J Respir Crit Care Med.2001; 163: 936-941.

11. Parr DG, Stoel BC, Stolk J, Stockley RA. Pattern of emphysema distribution in a-1-antitrypsin deficiency influences lung function impairment.American Journal of Respiratory Critical Care Medicine. 2004; 170 (11): 1172-1178

12 Dowson LJ, Guest PJ, Hill SL, Holder RL and Stockley RA. High-resolution computed tomography scanning in a1-antitrypsin deficiency: relationship to lung function and health status. Eur Respir J. 2001; 17: 1097-1104

13 Dawkins PA, Dowson LJ, Guest PJ, Stockley RA. Predictors of mortality in a1-antitrypsin deficiency.Thorax. 2003; 58: 1020-1026

14 Stolk J, Cooper BG, Stoel B, Rames A, Rutman O, Soliman S, Stockley RRetinoid treatment of Emphysema in Patients on the Alpha-1 International Registry. The REPAIR study: study design, methodology and quality control of study assessments. Ther Adv Respir Dis. 2010 Dec;4(6):319-32.

15. Newby PR, Crossley D, Crisford H, Stockley JA, Mumford RA, Carter RI, et al. A specific proteinase 3 activity footprint in α_1-antitrypsin deficiency. ERJ Open Res. 2019 Aug 5;5(3). pii: 00095-2019. doi: 10.1183/23120541.00095-2019

For peer review only - http://bmjopen.bmj.com/site/about/guidelines.xhtml

16. Miravitlles M, Dirksen A, Ferrarotti I, Koblizek V, Lange P, Mahadeva M, et al. European Respiratory Society statement: diagnosis and treatment of pulmonary disease in a1-antitrypsin deficiency. Eur Respir J. 2017; 50(5):1700610

17. Miravitlles M, Chorostowska-Wynimko J, Ferrarotti I, McElvaney NG, O'Hara K, Stolk J, et al. EARCO Clinical Research Collaboration; Members of the EARCO Clinical Research Collaboration. The European Alpha-1 Research Collaboration (EARCO): a new ERS Clinical Research Collaboration to promote research in alpha-1 antitrypsin deficiency. Eur Respir J. 2019 Feb 14;53(2).

18. Food and Drug Administration. Chronic obstructive pulmonary disease: developing drugs for treatment; draft guidance for industry. Fed Regist. 2016;81(98):31946.

For peer review only

Figure legends

Figure 1. The NIHR rare diseases AATD network
Individual centres are shown together with their principle investigators and link to the coordinating centre in Birmingham

Figure 2. Scatter plots of the relation between Lung function and CT densitometry analysis.
Each point represents data from a single patient. The correlation coefficient (Rho) is given for each analysis using the 2 best recognised parameters for emphysema on CT scan.

Figure 3. Box plots of CT parameters by GOLD stage.
Data is shown as box plot of IQR with median value indicated by the solid line, whiskers indicate 95% data range and outliers are indicated by open circles for each GOLD stage of severity.

Figure 4. QoL parameters determined by GOLD stage.
Data is shown as box plot of IQR with horizontal median line, whiskers indicate the 95% data range and outliers are shown as open circles. Patients with no airflow obstruction are shown as a separate group

Figure 5. Scatter plots to show the relation between QoL and CT analysis. Data is shown for individual patient CAT and total SGRQ scores related to emphysema parameters. The significance of the relationships is indicated (p)

For peer review only

For peer review only - http://bmjopen.bmj.com/site/about/guidelines.xhtml

Figure 1. The NIHR rare diseases AATD network
Individual centres are shown together with their principle investigators and link to the coordinating centre in Birmingham

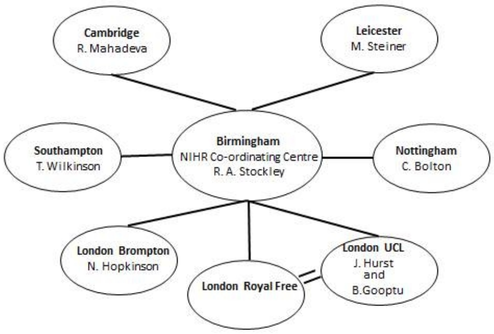

254x190mm (96 x 96 DPI)

Figure 2. Scatter plots of the relation between Lung function and CT densitometry analysis.
Each point represents data from a single patient. The correlation coefficient (Rho) is given for each analysis using the 2 best recognised parameters for emphysema on CT scan.

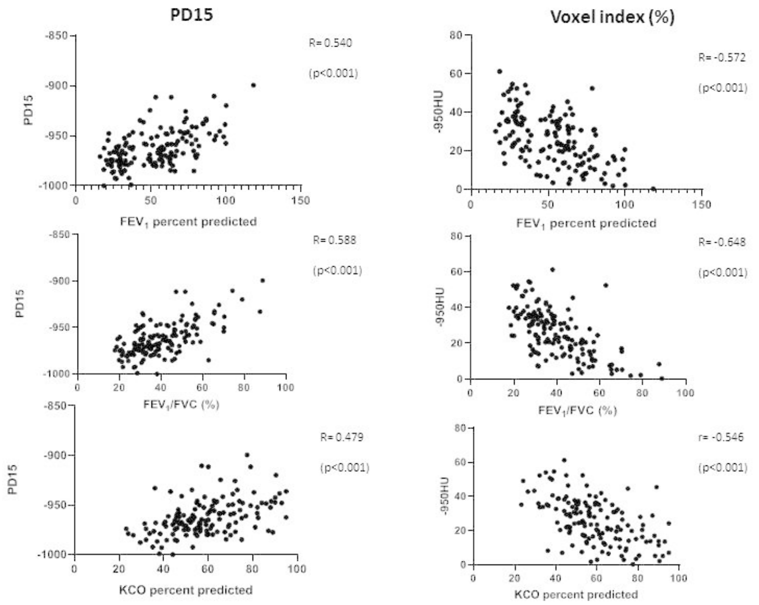

254x190mm (96 x 96 DPI)

Figure 3. Box plots of CT parameters by GOLD stage.
Data is shown as box plot of IQR with median value indicated by the solid line, whiskers indicate 95% data range and outliers are indicated by open circles for each GOLD stage of severity.

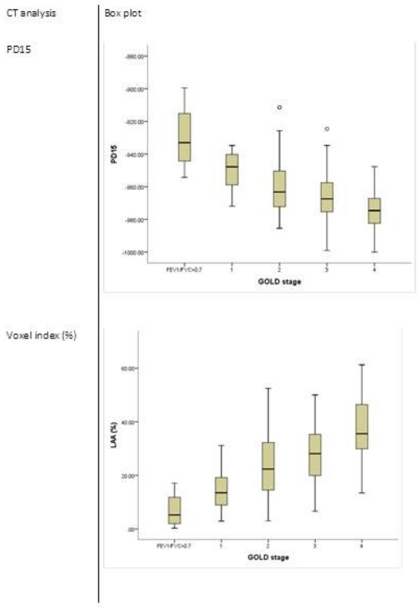

254x190mm (96 x 96 DPI)

Figure 4. QoL parameters determined by GOLD stage.
Data is shown as box plot of IQR with horizontal median line, whiskers indicate the 95% data range and outliers are shown as open circles. Patients with no airflow obstruction are shown as a separate group

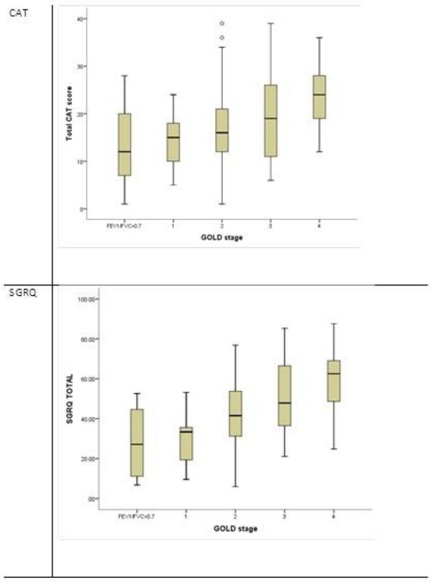

254x190mm (96 x 96 DPI)

Figure 5. Scatter plots to show the relation between QoL and CT analysis. Data is shown for individual patient CAT and total SGRQ scores related to emphysema parameters. The significance of the relationships is indicated (p)

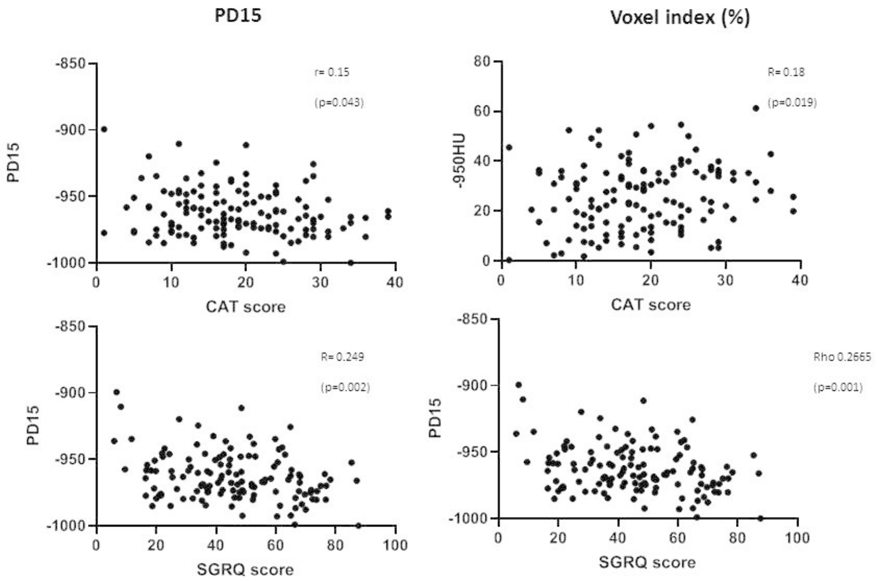

254x190mm (96 x 96 DPI)

Supplementary table and figures

The relationship between CT densitometry and clinical measures in Patients with Alpha one Antitrypsin Deficiency: the NIHR Rare diseases Translational Research Collaboration.

Diana Crossley[1], James Stockley[2], Charlotte E Bolton[3], Nicholas S Hopkinson[4], Ravi Mahadeva[5], Michael Steiner[6], Tom Wilkinson[7], John R Hurst[8] ,Bibek Gooptu[6,9] and Robert A Stockley[2].

Supplementary table. Summary of correlation co-efficients between Quality of Life (QoL) measures and lung function parameters.

	CAT	SGRQ
**FEV_1** (L)	-0.41*	-0.51*
**FEV_1** (%predicted)	-0.41*	-0.55*
**FVC** (L)	-0.27*	-0.32*
**FEV_1/FVC** (%)	-0.26*	-0.38*
**Kco** (mmol/min/.kPa/.L)	-0.12	-0.11
**Kco** (% predicted)	-0.26*	-0.23*
**RV** (L)	0.35*	0.46*
**TLC** (L)	-0.07	0.02
**RV/TLC** (%)	0.30*	0.32*

(*=p<0.01)

For peer review only

Supplemental Figure S1. Scatter plots to show the relation between SGRQ and FEV_1 percent predicted. Data is shown for individual patient total SGRQ scores related to their FEV_1.

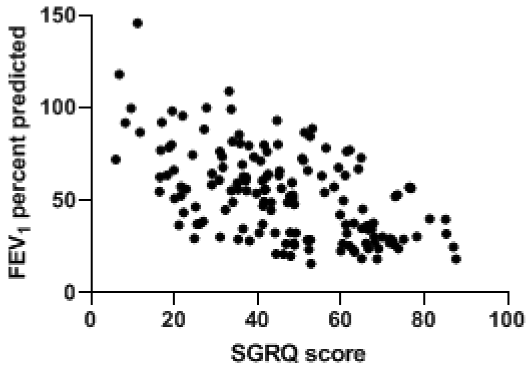

Supplemental figure S2. Scatter plots to show the relation between CAT and FEV_1 percent predicted. Data is shown for individual patient CAT scores related to their FEV_1.

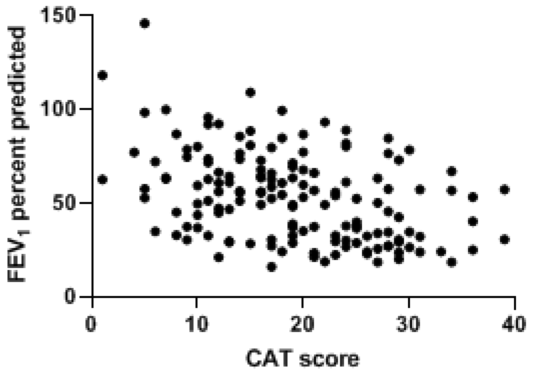

BMJ Open

The relationship of CT densitometry to Lung Physiological parameters and Health Status in Alpha-1 Antitrypsin deficiency; Initial report of a centralised data base of the NIHR Rare diseases Translational Research Collaborative.

Journal:	*BMJ Open*
Manuscript ID	bmjopen-2019-036045.R1
Article Type:	Original research
Date Submitted by the Author:	25-Apr-2020
Complete List of Authors:	Crossley, Diana ; Queen Elizabeth Hospital<br>Stockley, James; Queen Elizabeth Hospital<br>Bolton, Charlotte; University of Nottingham,<br>Hopkinson, Nicholas; Royal Brompton Hospital, Respiratory Muscle Lab<br>Mahadeva, Ravi; Addenbrookes Hospital<br>Steiner, michael ; Institute for Lung Health, NIHR Leicester Biomedical Research Centre – Respiratory, University of Leicester, Glenfield Hospital,<br>Wilkinson, Tom; University of Southampton, Faculty of Medicine<br>Hurst, John; UCL Medical School,, Academic Unit of Respiratory Medicine<br>Gooptu, Bibek; Institute for Lung Health, NIHR Leicester Biomedical Research Centre – Respiratory, University of Leicester, Glenfield Hospital<br>Stockley, Robert; Queen Elizabeth Hospital
**Primary Subject Heading**:	Respiratory medicine
Secondary Subject Heading:	Patient-centred medicine
Keywords:	Physiology < BASIC SCIENCES, GENERAL MEDICINE (see Internal Medicine), Thoracic medicine < INTERNAL MEDICINE, Computed tomography < RADIOLOGY & IMAGING, Emphysema < THORACIC MEDICINE, Chronic airways disease < THORACIC MEDICINE

SCHOLARONE™
Manuscripts

I, the Submitting Author has the right to grant and does grant on behalf of all authors of the Work (as defined in the below author licence), an exclusive licence and/or a non-exclusive licence for contributions from authors who are: i) UK Crown employees; ii) where BMJ has agreed a CC-BY licence shall apply, and/or iii) in accordance with the terms applicable for US Federal Government officers or employees acting as part of their official duties; on a worldwide, perpetual, irrevocable, royalty-free basis to BMJ Publishing Group Ltd ("BMJ") its licensees and where the relevant Journal is co-owned by BMJ to the co-owners of the Journal, to publish the Work in this journal and any other BMJ products and to exploit all rights, as set out in our licence.

The Submitting Author accepts and understands that any supply made under these terms is made by BMJ to the Submitting Author unless you are acting as an employee on behalf of your employer or a postgraduate student of an affiliated institution which is paying any applicable article publishing charge ("APC") for Open Access articles. Where the Submitting Author wishes to make the Work available on an Open Access basis (and intends to pay the relevant APC), the terms of reuse of such Open Access shall be governed by a Creative Commons licence – details of these licences and which Creative Commons licence will apply to this Work are set out in our licence referred to above.

Other than as permitted in any relevant BMJ Author's Self Archiving Policies, I confirm this Work has not been accepted for publication elsewhere, is not being considered for publication elsewhere and does not duplicate material already published. I confirm all authors consent to publication of this Work and authorise the granting of this licence.

For peer review only

The relationship of CT densitometry to Lung Physiological parameters and Health Status in Alpha-1 Antitrypsin deficiency; Initial report of a centralised data base of the NIHR Rare diseases Translational Research Collaborative.

Diana Crossley[1], James Stockley[2], Charlotte E Bolton[3], Nicholas S Hopkinson[4], Ravi Mahadeva[5], Michael Steiner[6], Tom Wilkinson[7], John R Hurst[8] ,Bibek Gooptu[6,9] and Robert A Stockley[2].

[1]University of Birmingham, College of Medical and Dental Sciences, Institute of Inflammation and Ageing, Centre for Translational Inflammation Research, Queen Elizabeth Hospital, Mindelsohn Way, Birmingham, B15 2WB, United Kingdom.

[2]University Hospitals Birmingham NHS Foundation Trust, Queen Elizabeth Hospital Birmingham, Mindelsohn Way, Edgbaston, Birmingham, B15 2GW, United Kingdom.

[3]Department of Respiratory Medicine, NIHR Nottingham BRC respiratory theme, School of Medicine, The University of Nottingham, City Hospital Campus, Hucknall Road, Nottingham. NG5 1PB, United Kingdom.[4]National Heart and Lung Institute, Imperial College, Royal Brompton Hospital Campus, Fulham Road, London, SW3 6NP, United Kingdom.

[5]Department of Medicine, Cambridge NIHR BRC, University of Cambridge, Cambridge, CB2 0QQ, United Kingdom.

[6]Institute for Lung Health, NIHR Leicester Biomedical Research Centre – Respiratory, University of Leicester, Glenfield Hospital, Leicester, United Kingdom.

[7] Respiratory BRU, University of Southampton, University Road, Southampton, SO17 1BJ, United Kingdom.

[8] Respiratory, Royal Free Campus, London, NW3 2PF.

[9] King's College London, Guy's Hospital Site, Great Maze Pond, London, SE1 9RT.

Corresponding Author: Professor Robert A Stockley, University Hospitals Birmingham NHS Foundation Trust, Queen Elizabeth Hospital Birmingham, Mindelsohn Way, Edgbaston, Birmingham, B15 2GW, United Kingdom. E-mail: r.a.stockley@bham.ac.uk; Tel: +44 121 371 6808. ORCID 0000-0003-3726-1207

For peer review only

Abstract (word count 299)

Objectives

To establish a data base network for the study of alpha-1 antitrypsin deficiency (AATD) and compare the results to computed tomographic (CT) lung density as the most direct measure of emphysema.

Design

A central electronic data base was established to permit the upload of anonymised patient data from remote sites. Prospectively collected CT data was recorded onto disc, anonymised, analysed at the coordinating centre and compared with the clinical features of the disease.

Setting

Tertiary referral centres with expertise in the management of AATD focused on academic Biomedical Research Units and Wellcome Clinical Research Facilities.

Participants.

Data was collected from 187 patients over 1 year from 8 UK academic sites. This included patient demographics, post bronchodilator physiology, health status and computed tomography. Analysis was undertaken at the coordinating centre in Birmingham.

Results.

Patient recruitment in the 12 months reached 94% of target (set at 200) covering the whole spectrum of the disease from those with normal lung function to very severe chronic obstructive lung disease. CT scan suitable for analysis was available from 147 (79%) of the patients. CT density, analysed as the threshold for the lowest 15% of lung voxels, showed statistically significant relationships with the objective physiological parameters of lung function as determined by spirometric GOLD severity staging ($p< 0.001$) and carbon monoxide gas transfer ($p<0.01$). Density also correlated with subjective measures of quality of life ($p=0.02$).

Conclusions.

Establishment of the network for data collection and its transfer was highly successful facilitating future collaboration for the study of this rare disease and its management. CT densitometry correlated well with the objective clinical features of the disease supporting its role as the specific marker of the associated emphysema and its severity. Correlations

with subjective measures of health, however, were generally weak indicating that other factors play a role.

Strengths and limitations of this study

The strengths of the project has been the design and delivery of a centralised registry for alpha-1 antitrypsin deficiency enabling the whole spectrum of the disease to be characterised especially including those with more severe disease who are less able to travel long distances to single National centres of excellence.

Recruitment and data collection was rapid and 94% of target.

The study provided further validation of the specificity of quantitative analysis of lung density for the assessment of emphysema in this "rare" disease.

The limitations of the study included the slight shortfall in patient recruitment over the 1 year target and slightly incomplete post bronchodilator physiological data entry.

A reduced number of CT scans were analysed because of recent routine scans performed under different parameters. Although all patients filled in one of the health status tools this was not 100% for both.

For peer review only

Key Words.
Alpha-1antitrypsin deficiency, Emphysema, Computed Tomography lung physiology, Health status

INTRODUCTION

Alpha one antitrypsin deficiency (AATD) affects around 1 in 2500 individuals in the UK and increases the susceptibility to develop chronic obstructive pulmonary disease (COPD)

with an early onset emphysema dominant phenotype and adult onset liver cirrhosis. AAT is the main plasma serine proteinase inhibitor and is predominantly made and secreted by the liver. It enters the tissues by simple diffusion especially in the presence of inflammation where it regulates the local activity of neutrophil proteinases (1). This is important in delicate tissue structures such as the alveolar region of the lung where uncontrolled serine proteinases can destroy connective tissues leading to alveolar destruction and the development of emphysema (2).

The study and treatment of rare diseases presents many problems including patient identification, referral to centres with relevant expertise, consistent demographic characterisation and importantly the design and delivery of appropriately powered research studies and clinical trials. With this in mind the UK National Institute for Health Research commissioned a series of projects in 2014 to develop networks for the study and deep phenotyping of rare diseases with the longer term aim of providing a consistent structure to facilitate collaboration between groups and with industrial partners to further research and therapeutic development.

In 1996 the ADAPT programme (Antitrypsin Deficiency and Programme for Treatment) was established in Birmingham as an investigator led, industry and research grants funded project in order to study AATD and its natural history. The programme depended on referrals, mainly from secondary care centres in the UK. Patients were invited to visit Birmingham on an annual basis for assessment as part of a clinical/ research programme (LREC 3359a). However, although successfully collating data on over 1000 deficient patients, those seen may be affected by bias due to referral patterns as well as to issues of geography and transport difficulties for annual visits. The latter issues may be especially relevant for the most severely affected patients. The ADAPT cohort may therefore underestimate the true impact of especially the severe end of the disease spectrum. In addition the evolution of a single centre impairs delivery of near patient care, clinical trials and iterative thought which can be handicapped by the absence of other local centres of excellence more convenient for patient attendance, management and especially, recruitment/participation in research and clinical trials.

The current article describes the AATD collaborative network funded by the National Institute of Health Research to establish a patient cohort for rare diseases collated over 1 year in the UK. In particular we wished to recruit patients with a wide range of severity, with the most severe being recruited to the closest specialist centre to minimise the difficulty of travel. In particular we wished to assess lung densitometry as the most direct

measure of emphysema and physiological impairment and patients perception of their health status.

MATERIALS AND METHODS

Birmingham acted as the coordinating centre for the National Institute for Health Research Rare Diseases Translational Research Collaboration in AATD. Links were made with academic Biomedical Research Units where a specialist with expertise in clinical trials and an interest in AATD had been identified. The network had an initial target of 200 patients with AATD and the PiZZ genotype recruited from Birmingham, Nottingham, London Brompton, London UCL/Royal Free Hospital, Cambridge, Southampton and Leicester (see figure 1). The aim was to recruit 100 patients from Birmingham with mild and moderate disease and 100 patients with severe and very severe disease (as defined by the FEV_1) from the other centres such that the distance these patients had to travel was less than attending a single central facility. A standard protocol was provided to all centres based on that used in all previous publications from the core Birmingham centre and consisted of general demographic data required for in depth patient characterisation that would provide the basis of patient identification for inclusion in subsequent clinical trials using National standard operating procedures for lung function and specific parameters for CT acquisition.

Patients were recruited sequentially from the individual centre clinics according to the spirometric criteria outlined above. All patients gave informed consent and the study was approved for all sites by the local ethics committee (West Midlands – South Birmingham Research Ethics Committee, LREC 3359).

All subjects underwent full clinical examination, were scheduled to undergo full post bronchodilator lung function testing (according to the Association of Respiratory Technology/British Thoracic Society guidelines for quality control) including spirometry gas transfer and lung volumes, determination of current health status using well established tools (St George's Respiratory Questionnaire; SGRQ and the COPD assessment tool; CAT). Where routine high resolution Computed Tomography had not been undertaken within the previous 2 years, a scan on full inspiration was taken using the following fixed criteria:- subjects were scanned by spiral multi-slice CT of the chest in the supine position within 4 hours of administration of a short-acting bronchodilator. A low radiation dose (140kvp) was used, the slice thickness 5mm and increment 2.5mm,

and a soft reconstruction algorithm (B30f) applied. The anonymised data was subsequently transferred to Birmingham on disc for quantitative analysis using Pulmo-CMS (Medis Medical Imaging, Leiden, Netherlands courtesy of Berend Stoel). Measurements for voxel index (% Voxels less dense than -950HU) and Perc15 (density threshold of the lowest 15% of the voxels) were assessed for the whole lung such that greater Voxel index or lower Perc (PD)15 indicated a greater amount of emphysema.

Patient and Public Involvement.

The project was approved by 5 patients who completed a Traffic Light System (TLS) research proposal review form to give their opinion on the research project and the significance of its impact on clinical practice. A further patient representative was a formal member of the steering committee. The information will be part of future presentation to the patient group and summarised for dissemination in their annual newsletter once accepted for publication. Results will also be presented at international specialty meetings. This project represented a first step towards achieving the long term aims expressed by patients as part of the European lung Foundation survey of alpha-1 antitrypsin deficiency.

Statistical Analysis

Baseline characteristics are presented as median and interquartile range (IQR). The Pearson's or Spearman rank correlation coefficient between CT density and PFTs was calculated. Analysis of variance (ANOVA) or Kruskall-Wallis was used to assess differences between grouped data. Normality of the data was tested using the Shapiro Wilk test, and the level of significance was conventionally determined at $p<0.05$.

RESULTS

Baseline characteristics

There were 187 patients recruited to the study across the eight sites within the 1 year recruitment time. Of these 84 with severe and very severe COPD were recruited from the satellite sites and 94 mild and moderate disease recruited by Birmingham patients as well

as 9 local patients with very severe disease. Confirmed post bronchodilator spirometry was available for 185 patients and carbon monoxide gas transfer (single breath) for 181. Quantitative CT scan was obtained for 147 patients and all patients completed quality of life metrics (SGRQ and/or CAT) although only 158 patients completed both.

The baseline characteristics are summarised in table 1. Data are presented as median and interquartile range to characterise the spread of patients studied. The median age was 60 years and the median post bronchodilator FEV_1 for the cohort was 51.1% predicted for age, sex, height and ethnicity (3). Of these 44 (24%) patients, (recruited from the peripheral sites) were graded as GOLD stage 4 at the time of study (4). 9 patients with AATD (5%) had an $FEV_1/FVC > 0.7$. The median CAT score for the whole group was 18.9 (12.0-25.0) and the median total SGRQ score was 45.2 (33.3-62.1).

Smoking history information was available for 178 patients. 79 were lifelong never smokers but all patients with a smoking history had ceased for more than 12 months at the time of recruitment. The median smoking history for the ex-smokers (n=99) was 17 pack years (IQR 8-27). None of the patients were receiving AAT augmentation therapy as it remains unavailable for management of Emphysema in the UK.

Cross sectional correlations

Lung function and CT densitometry

The relationship between CT density and lung function is shown in figure 2. There were significant correlations between both lung density parameters and the physiological parameters shown (p<0.001 all comparisons). Significant differences were also seen for CT density measurements as both the voxel index (which quantifies the proportion of low density voxels below -950 HU) and PD15 (which quantifies the threshold below which 15% of the overall voxels are sited), between each of the GOLD severity stages (p<0.001) (figure 3). There was no correlation between RV/TLC and any of the densitometry parameters.

Table 1. Baseline Characteristics Table. Data is shown as median and interquartile ranges together with the number of patients where data was collected. All subjects were either never smokers or had ceased at least 1 year before the time of assessment. CAT score is only given for those where the total SGRQ score was also available.

Clinical Variable	Number of	Median (IQR)

For peer review only - http://bmjopen.bmj.com/site/about/guidelines.xhtml

	subjects	
Age (yrs)	187	60.1 (50.9-66.9)
Sex	86M 101F (46%M, 54% F)	
Pack years for ex-smokers	99	17 (8-27)
FEV_1(L)	185	1.4 (0.9-1.9)
FEV_1percent predicted	185	51.1 (30.5-67.5)
FVC(L)	185	3.6 (2.9-4.8)
FVC percent predicted	185	105.7 (88.2-123.4)
FEV_1/FVC (%)	185	36.9 (28.5-49.8)
Kco percent predicted	181	56.3 (46.0 - 69.6)
RV/TLC (%)	140	41.5 (33.0-52.7)
Voxel index -950 HU (%)	147	24.5 (15.4-35.2)
Perc15 (HU)	147	-965.2 (-974.8—950.7)
Total CAT score	158	18.9 (12.0-25.0)
Total SGRQ score	158	45.2 (33.3-62.1)

Following adjustment of all these relationships for age, sex and pack years, the relationship between density as measured by PD15 and voxel index remained significant (p<0.001) with no evidence of collinearity (see table 2).

Table 2. Multivariate linear regression models between CT density and Lung function.

Data is shown for the regression analysis of both CT parameters and baseline lung function. "pp" is the percent predicted

CT density	Lung function parameter	Unstandardised co-efficient	Standard error
PD15	FEV_1pp	0.63	0.1 (p<0.001)
	FEV_1/FVC (%)	0.94	0.1 (p<0.001)

	Kcopp	0.55	0.1(p<0.001)
Voxel index (%)	FEV_1pp	-0.48	0.1 (p<0.001)
	FEV_1/FVC (%)	-0.67	0.1 (p<0.001)
	Kcopp	-0.5	0.8 (p<0.001)

Quality of life measures and Lung Function

Within the cohort, the CAT test scores ranged between 1 and 39/40 (median=18.9; IQR 12-25) and related to disease severity as defined by the GOLD stage and summarised in figure4 (p<0.0001). The median SGRQ was 45.2 (33.3-62.1) and also related to the GOLD stage (p<0.001) shown in figure 4. (p<0.001). The median and IQR for CAT and SGRQ in each GOLD stage is summarised in table 3.

Table 3. Quality of Life data across each GOLD stage (4). The number of patients for each GOLD stage is shown together with the median and IQR data for the CAT and SGRQ total score.

GOLD Stage	Patient number	CAT median (IQR)	Patient number	SGRQ median (IQR)
FEV1/FVC>0.7	9	12 (6-21)	9	27.1 (9.7-48.0)
1	13	15 (9-21)	12	33.4 (19.3-35.7)
2	63	16 (12-21)	63	41.5 (30.9-55.4)
3	39	19 (11-25)	39	48.8 (36.5-67.3)
4	53	20 (13.5-27)	33	62.0 (36.5-67.3)

There were significant correlations (Supplemental Table 1) between both QoL measures and spirometry, as measured by FEV_1 (percent predicted), FVC (percent predicted) , FEV_1/FVC (%), and with gas transfer co-efficient, Kco (percent predicted) and gas trapping as measured by RV/TLC % (p<0.01 all comparisons). Supplemental figures 1 and 2 summarise the correlations for SGRQ and CAT with FEV_1 (% predicted)

Quality of life measures and CT density

Both Quality of life measures were worse the greater the emphysema as quantified by the

For peer review only - http://bmjopen.bmj.com/site/about/guidelines.xhtml

lung density. There was a significant relationship between voxel index and CAT (r= 0.18, p=0.019) and a weaker relationship with PD15 (r=0.15, p=0.043) (figure 5). Total SGRQ correlated significantly with both measures of CT density, although again the relationship was weak (r^2<0.1) (see figure 5).

Discussion

The current paper reports demographic data collated from a unique NIHR funded project to establish collaborative cohorts of patients with rare diseases focused on academic Biomedical Research Units and Wellcome Clinical Research Facilities. The main purpose of this project was to recruit 200 patients (none of whom were receiving augmentation therapy) with AATD (PiZZ genotype) to a central data base over a 1 year period as a basis for ongoing collaborative research. The aim of the collaborative was to recruit and characterise patients with AATD in depth across centres with a wide spectrum of severity. Importantly we wished to include those with severe and very severe disease (GOLD stage 3 and 4) as quantified by baseline FEV_1 (4) in the satellite centres local to their dwelling who are more likely to refrain from travel to a more central facility. Of note the data for the very severe patients produced similar values and demographic relationships to those seen historically in the single centre ADAPT programme in Birmingham.

We have confirmed the feasibility of this approach with recruitment from multiple academic centres in this limited time period reaching 94% of the target. Of these 98% had confirmed post bronchodilator spirometry (in 2 it had not been documented) , 92% were able to complete gas transfer measurements and 85% had both SGRQ and CAT measurement of health status although most patients (95%) had CAT recorded. CT scans were a unique feature of the UK patients and were performed prospectively at baseline using a fixed protocol and copied anonymously on to disc for analysis in Birmingham. This resulted in a total of 147 scans available and suitable for analysis (79% of patients) enabling the relationships to clinical parameters to be explored.

The patients showed a wide spectrum of disease including 53 patients local to the participating centres classified as GOLD stage 4. Health status showed a relationship to

spirometric severity with worsening for each stage although (as expected) with a wide range and major group overlap as reported from the larger ADAPT data base (5) and other studies of non AATD COPD (6). Importantly the patients in GOLD stage 4 had a median total SGRQ score 62.0 (IQR 36.5-67.3) which was similar to that for 51 stage 4 patients recruited previously to the ADAPT data base (extracted from ref 5) with a median value of 59.1 (IQR 51.8-72.0). This suggests that our previous data was not influenced by acquisition bias as a result of travel to a single centre being influenced by distance and/or patient health. In addition it indicates that GOLD 4 patients can be readily recruited from multiple sites for research, audit and appropriate clinical trials purposes.

Relationship of lung densitometry (the most direct measure of pathological emphysema) to objective parameters of lung function was similar to that seen in other studies (7,8) but better for gas transfer than spirometry (consistent with the former being a more direct measure of alveolar dysfunction as seen in emphysema) and certainly better than the weak relationship to air trapping (RV/TLC) and the subjective health status measures. COPD even in AATD is a multifaceted condition although classically characterised by the presence of panacinar emphysema localised to the lung bases as the dominant pathological phenotype. Lung densitometry is the most direct measure of this process and its severity as demonstrated pathologically (9). All other measures are indirect surrogate markers of emphysema and although densitometry in AATD relates to exercise capacity (10) spirometry and gas transfer (11) and health status (12) it remains the best independent predictor of mortality in this disease (13). In addition it is the most sensitive measure of emphysema progression in AATD (14) and hence its support as the primary outcome in clinical trials enabling such studies to be sufficiently powered for such a rare disease. CT has not been routinely undertaken as part of management of AATD patients largely on the basis of cost and radiation exposure. However, the radiation exposure protocol for densitometry is less than that for high resolution scans and is specific for the pathological phenotype of the COPD in AATD (namely the emphysema). Indeed the recent European Respiratory Society strategy document on AATD has confirmed it as part of the assessment for all such patients and that referral to centres with a major interest in AATD should be included in patient management (15) The relationships of CT densitometry to more recognised parameters of COPD severity and mortality are no worse and certainly better (specifically mortality) than some of the more conventional markers of the disease although still only weakly related to the subjective interpretation

For peer review only - http://bmjopen.bmj.com/site/about/guidelines.xhtml

of the patients health.

The study was successful in enabling collaboration between academic centres by providing recruitment and in depth phenotyping of a group of patients with AATD and a wide range of physiological impairment to an agreed standard. All subjects also had plasma taken and stored for the development and validation of assays to monitor serine proteinase footprints (16) and other disease specific biomarkers. In addition the network provides baseline cohort data for long term follow up and importantly a well characterised patient data base for collaboration with industry (one of the key aims for the NIHR programme). Furthermore it conforms to the key elements outlined by the European Respiratory Society strategy document for the management of AATD (15); namely establishment of registries, comprehensive assessment and review by specialists with an interest in AATD. It also provides the background for linking with the European Union rare diseases collaborative and the European Alpha-1 Research Collaboration (EARCO) (17).

Nevertheless the study has some limitations. Several of the patients had undergone CT scanning as part of their routine clinical assessment prior to the establishment of the collaborative and hence it was not deemed ethical to repeat the procedure in the short term for densitometry analysis as part of a phenotyping study. However the parameters are set for all future scans in this patient population both at baseline and for monitoring progression. The documentation of health status was not perfectly consistent although either the SGRQ or CAT was performed in all subjects. Only 85% had the SGRQ documented whereas 95% had the CAT documented which may reflect the latter's ease of administration and practical clinical utility (4). Our analysis reported here only included those patients in whom both had been measured. Currently only the SGRQ has been accepted as a validated tool for patient reported outcomes in clinical trials (18) whereas the CAT has become an accepted measure in patient management as supported by GOLD(4) and is often also reported in clinical studies and trials. However, such measures of health status are best used to indicate a noticeable change from baaseline for therapies that provide an initial and detectable impact such as bronchodilators and not therapies that modify slow progression of chronic diseases such as COPD and emphysema (5).Therefore although densitometry relates to both FEV_1 and health status

(though poorly for the latter) evidence of progression and response to treatment in AATD should currently depend on CT scans as the most direct and sensitive marker of emphysema and its progression as confirmed recently in a placebo controlled trial of the benefit of augmentation therapy (19)..

In summary the project successfully recruited to 94% of target in the one year time frame and the majority underwent deep phenotyping to a set standard for future patient care and assessment consistent with the view of the European Respiratory Society strategy document for AATD (15). The collaboration and expertise of participants provides a firm structure for future management of AATD in the UK. Importantly it provides validation that previous patient assessment as part of the ADAPT programme reflects patients across the spectrum of disease severity and not a patient acquisition bias related to distance or travel. Furthermore it demonstrates an intercalated network that can harmonise activity and research outcomes using standard methodologies. These points give reassurance that patient monitoring and care can be provided at multiple sites to the same standard, factors that are both critical in patient convenience and reassurance, as expressed by patients in the recent ERS AATD strategy document (15). Importantly it provides validation of CT densitometry for characterisation of the pathological nature and severity of the disease and places this in perspective with other measures routinely used for monitoring. Because of its specificity it should become an essential part of AATD assessment (15). Finally it provides an essential ready made resource for the delivery of research, clinical studies and trials especially for the pharmaceutical industry and a UK resource for patient/specialist research collaboration.

Author Contributions

The project concept was developed by RAS. JAS, CEB, NSH, JRH, BG, RM, MS, TAW and RAS established the network as part of the NIHR rare diseases call, collated clinical, physiological and radiological data across each contributing organisation. DC analysed the quantitative CT scans and the related data. DC drafted and refined the manuscript with RAS. All authors reviewed, modified and approved the final manuscript. None of the authors have financial relationships with any organisations in the previous three years

that might have an interest in the submitted work; no other relationships or activities that could appear to have influenced the submitted work. However, RAS sits on several advisory boards for pharmaceutical companies with or developing treatments for alpha-one antitrypsin deficiency and NSH is currently recruiting to a phase 2 study in these patients sponsored by Mereo Biopharma.

Access to data

Anonymised data is stored on the central data base in Birmingham and site specifically at the collaborative sites. Access to data can be obtained by approval of the registry holders following application to the senior author

Acknowledgements: We would like to thank Anita Pye, the study coordinator for her assistance and support and Mr J Beggs the patient representative on the Steering committee.

The Research and Network was funded by The NIHR rare diseases TRC and coordinated from the National Institute for Health Research (NIHR)/Wellcome Trust Birmingham Clinical Research Facility,. *NIHR Nottingham BRC respiratory theme , Cambridge NIHR BRC ,NIHR Leicester BRC – respiratory theme, Respiratory BRU Southampton, NIHR Respiratory BRU at Royal Brompton and Harefield NHS Foundation Trust and Imperial College, London*

The views expressed are those of the authors(s) and not necessarily those of the NHS, the NIHR or the Department of Health.

References

1. Stockley RA.. The measurement of soluble proteins in lung secretions. Thorax. 1984; 39: 241-247.

2. Stockley RA. Alpha-1-antitrypsin and the pathogenesis of emphysema. Lung. 1987; 165: 61-77.

3. Quanjer PH, Tammeling GJ, Cotes JE, Pedersen OF, Peslin R, Yernault JC. Lung volumes and forced ventilatory flows. Eur Respir J. 1993;6 Suppl 16:5-40.

4. Vogelmeier CF, Criner GJ, Martinez FJ, Anzueto A, Barnes PJ, Bourbeau J, et al.A Global Strategy for the Diagnosis, Management, and Prevention of Chronic Obstructive Lung Disease 2017 Report. GOLD Executive Summary. Am J Respir Crit Care Med. 2017 Mar 1;195(5):557-582

5. Stockley RA, Edgar RG, Starkey S, Turner AM. Health status decline in α-1 antitrypsin deficiency: a feasible outcome for disease modifying therapies? Respir Res. 2018; Jul 20;19(1):137.

6. Tabberer M, Benson VS, Gelhorn H, Wilson H, Karlsson N, Müllerova H, et al. The COPD Biomarkers Qualification Consortium Database: Baseline Characteristics of the St George's Respiratory Questionnaire Dataset. Chronic Obstr Pulm Dis. 2017 Mar 13;4(2):112-123

7. Kim SS, Seo JB, Lee HY, et al. Chronic obstructive pulmonary disease: lobe-based visual assessment of volumetric CT by using standard images-comparison with quantitative CT and pulmonary function test in the COPDGene study. Radiology. 2013;266(2):626–635

8. Barjaktarevic I, Springmeyer S, Gonzalez X, Sirokman W, Coxson HO, Cooper CB. Diffusing capacity for carbon monoxide correlates best with tissue volume from quantitative CT scanning analysis. Chest. 2015;147(6):1485–1493.

9. Gevenois PA, de Maertelaer V De Vuyst P, Zanen J and Yernault JC.Comparison of computed density and macroscopic morphometry in pulmonary emphysema. Am J Resp Crit Care Med. 1995;152:653-657.

10. Dowson LJ, Newal Cl, Guest PJ, Hill SL and Stockley RA. Exercise capacity predicts health status in α1-antitrypsin deficiency. Am J Respir Crit Care Med.2001; 163: 936-941.

11. Parr DG, Stoel BC, Stolk J, Stockley RA. Pattern of emphysema distribution in a-1-antitrypsin deficiency influences lung function impairment.American Journal of

For peer review only

Respiratory Critical Care Medicine. 2004; 170 (11): 1172-1178

12 Dowson LJ, Guest PJ, Hill SL, Holder RL and Stockley RA. High-resolution computed tomography scanning in a1-antitrypsin deficiency: relationship to lung function and health status. Eur Respir J. 2001; 17: 1097-1104

13 Dawkins PA, Dowson LJ, Guest PJ, Stockley RA. Predictors of mortality in a1-antitrypsin deficiency.Thorax. 2003; 58: 1020-1026

14 Stolk J, Cooper BG, Stoel B, Rames A, Rutman O, Soliman S, Stockley R. methodology and quality control of study assessments.Retinoid treatment of Emphysema in Patients on the Alpha-1 International Registry. The REPAIR study: study design, Ther Adv Respir Dis. 2010 Dec;4(6):319-32.

15 Miravitlles M, Dirksen A, Ferrarotti I, Koblizek V, Lange P, Mahadeva M, et al. European Respiratory Society statement: diagnosis and treatment of pulmonary disease in a1-antitrypsin deficiency. Eur Respir J. 2017; 50(5):1700610

16 Newby PR,Crossley D, Stockley JA, Mumford RA, Carter RI, et al. A specific proteinase 3 activity footprint in α_1-antitrypsin deficiency. ERJ Open Res. 2019 Aug 5;5(3). pii: 00095-2019. doi: 10.1183/23120541.00095-2019

17. Miravitlles M, Chorostowska-Wynimko J, Ferrarotti I, McElvaney NG, O'Hara K, Stolk J, et al. EARCO Clinical Research Collaboration; Members of the EARCO Clinical Research Collaboration. The European Alpha-1 Research Collaboration (EARCO): a new ERS Clinical Research Collaboration to promote research in alpha-1 antitrypsin deficiency. Eur Respir J. 2019 Feb 14;53(2).

18. Food and Drug Administration. Chronic obstructive pulmonary disease: developing drugs for treatment; draft guidance for industry. Fed Regist. 2016;81(98):31946.

19.Chapman KR, Burdon JG, Piitulainen E, Sandhaus RA, Seersholm N, Stocks JM, Stoel BC, Huang L, Yao Z, Edelman JM, McElvaney NG, Group RTS. Intravenous

For peer review only

augmentation treatment and lung density in severe alpha1 antitrypsin deficiency (RAPID): a randomised, double-blind, placebo controlled trial. Lancet 2015; 386: 360-368.

Figure legends

Figure 1. The NIHR rare diseases AATD network
Individual centres are shown together with their principle investigators and link to the coordinating centre in Birmingham

Figure 2. Scatter plots of the relation between Lung function and CT densitometry analysis.
Each point represents data from a single patient. The correlation coefficient (Rho) is given for each analysis using the 2 best recognised parameters for emphysema on CT scan.

Figure 3. Box plots of CT parameters by GOLD stage.
Data is shown as box plot of IQR with median value indicated by the solid line, whiskers indicate 95% data range and outliers are indicated by open circles for each GOLD stage of severity.

Figure 4. QoL parameters determined by GOLD stage.
Data is shown as box plot of IQR with horizontal median line, whiskers indicate the 95% data range and outliers are shown as open circles. Patients with no airflow obstruction are shown as a separate group

Figure 5. Scatter plots to show the relation between QoL and CT analysis. Data is shown for individual patient CAT and total SGRQ scores related to emphysema parameters. The significance of the relationships is indicated (p)

For peer review only

Figure 1. The NIHR rare diseases AATD network
Individual centres are shown together with their principle investigators and link to the coordinating centre in Birmingham

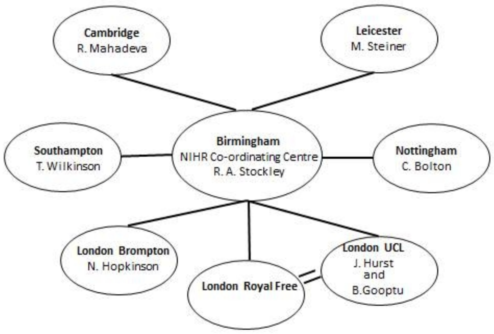

254x190mm (96 x 96 DPI)

Figure 2. Scatter plots of the relation between Lung function and CT densitometry analysis.
Each point represents data from a single patient. The correlation coefficient (Rho) is given for each analysis using the 2 best recognised parameters for emphysema on CT scan.

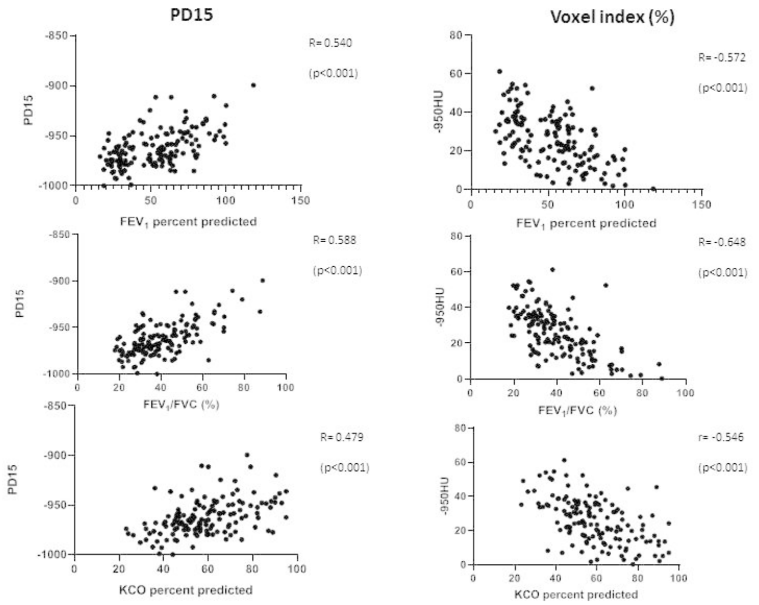

254x190mm (96 x 96 DPI)

Figure 3. Box plots of CT parameters by GOLD stage.
Data is shown as box plot of IQR with median value indicated by the solid line, whiskers indicate 95% data range and outliers are indicated by open circles for each GOLD stage of severity.

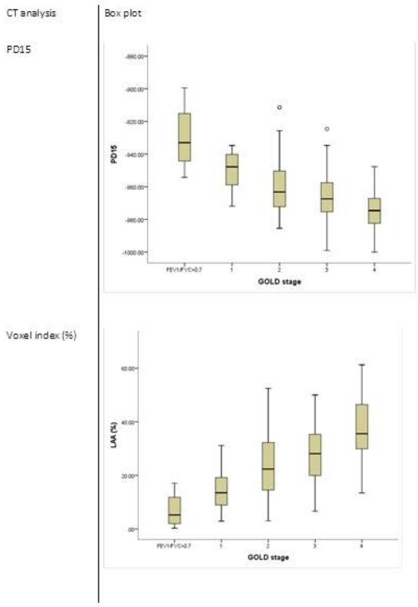

254x190mm (96 x 96 DPI)

Figure 4. QoL parameters determined by GOLD stage.
Data is shown as box plot of IQR with horizontal median line, whiskers indicate the 95% data range and outliers are shown as open circles. Patients with no airflow obstruction are shown as a separate group

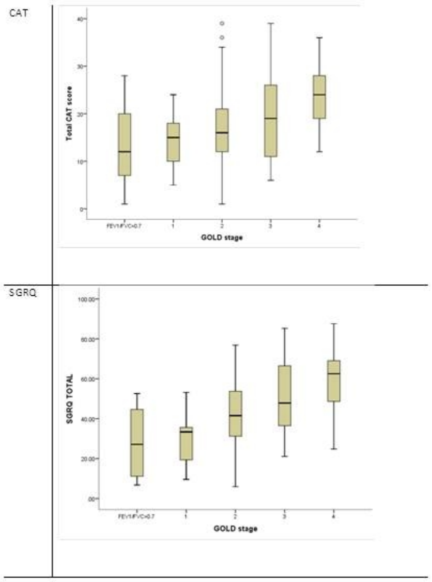

254x190mm (96 x 96 DPI)

Figure 5. Scatter plots to show the relation between QoL and CT analysis. Data is shown for individual patient CAT and total SGRQ scores related to emphysema parameters. The significance of the relationships is indicated (p)

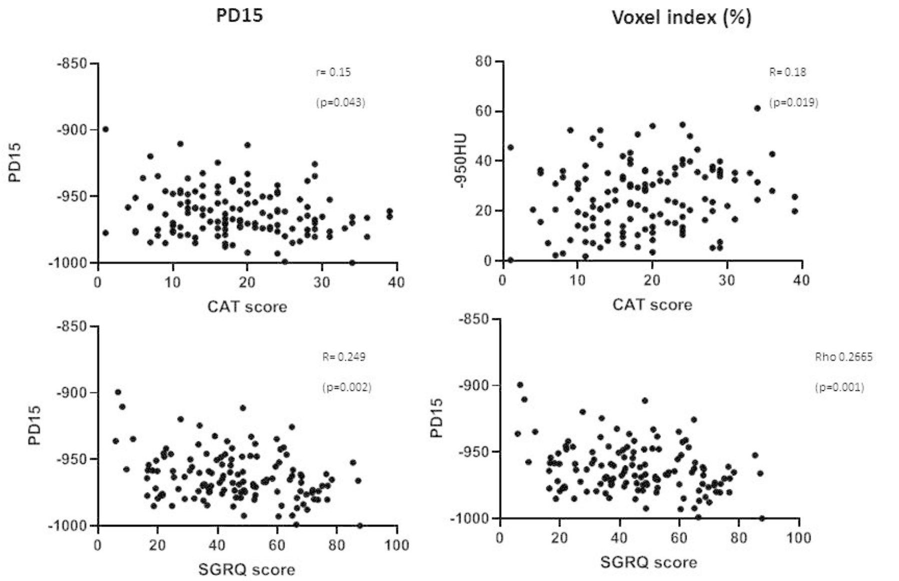

254x190mm (96 x 96 DPI)

Supplementary table and figures

The relationship between CT densitometry and clinical measures in Patients with Alpha one Antitrypsin Deficiency: the NIHR Rare diseases Translational Research Collaboration.

Diana Crossley[1], James Stockley[2], Charlotte E Bolton[3], Nicholas S Hopkinson[4], Ravi Mahadeva[5], Michael Steiner[6], Tom Wilkinson[7], John R Hurst[8] ,Bibek Gooptu[6,9] and Robert A Stockley[2].

Supplementary table. Summary of correlation co-efficients between Quality of Life (QoL) measures and lung function parameters.

	**CAT**	**SGRQ**
**FEV_1** (L)	-0.41*	-0.51*
**FEV_1** (%predicted)	-0.41*	-0.55*
**FVC** (L)	-0.27*	-0.32*
**FEV_1/FVC** (%)	-0.26*	-0.38*
**Kco** (mmol/min/.kPa/.L)	-0.12	-0.11
**Kco** (% predicted)	-0.26*	-0.23*
**RV** (L)	0.35*	0.46*
**TLC** (L)	-0.07	0.02
**RV/TLC** (%)	0.30*	0.32*

(*=p<0.01)

For peer review only

Supplemental Figure S1. Scatter plots to show the relation between SGRQ and FEV_1 percent predicted. Data is shown for individual patient total SGRQ scores related to their FEV_1.

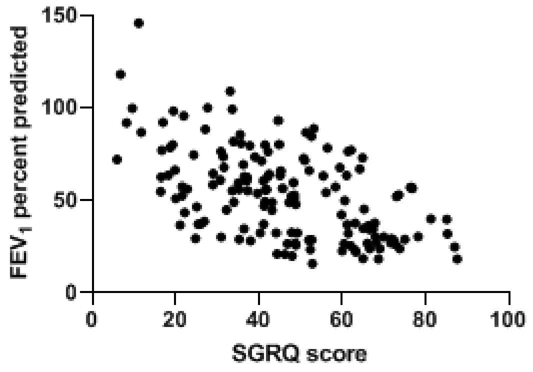

Supplemental figure S2. Scatter plots to show the relation between CAT and FEV_1 percent predicted. Data is shown for individual patient CAT scores related to their FEV_1.

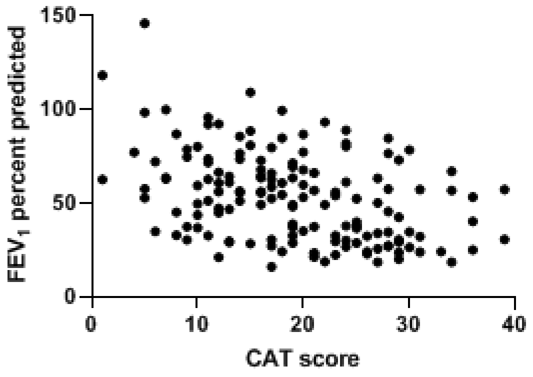

BMJ Open

The relationship of CT densitometry to Lung Physiological parameters and Health Status in Alpha-1 Antitrypsin deficiency; Initial report of a centralised data base of the NIHR Rare diseases Translational Research Collaborative.

Journal:	*BMJ Open*
Manuscript ID	bmjopen-2019-036045.R2
Article Type:	Original research
Date Submitted by the Author:	20-May-2020

Complete List of Authors:	Crossley, Diana ; Queen Elizabeth Hospital<br>Stockley, James; Queen Elizabeth Hospital<br>Bolton, Charlotte; University of Nottingham,<br>Hopkinson, Nicholas; Royal Brompton Hospital, Respiratory Muscle Lab<br>Mahadeva, Ravi; Addenbrookes Hospital<br>Steiner, michael ; Institute for Lung Health, NIHR Leicester Biomedical Research Centre – Respiratory, University of Leicester, Glenfield Hospital,<br>Wilkinson, Tom; University of Southampton, Faculty of Medicine<br>Hurst, John; UCL Medical School,, Academic Unit of Respiratory Medicine<br>Gooptu, Bibek; Institute for Lung Health, NIHR Leicester Biomedical Research Centre – Respiratory, University of Leicester, Glenfield Hospital<br>Stockley, Robert; Queen Elizabeth Hospital

**Primary Subject Heading**:	Respiratory medicine
Secondary Subject Heading:	Patient-centred medicine
Keywords:	Physiology < BASIC SCIENCES, GENERAL MEDICINE (see Internal Medicine), Thoracic medicine < INTERNAL MEDICINE, Computed tomography < RADIOLOGY & IMAGING, Emphysema < THORACIC MEDICINE, Chronic airways disease < THORACIC MEDICINE

SCHOLARONE™
Manuscripts

I, the Submitting Author has the right to grant and does grant on behalf of all authors of the Work (as defined in the below author licence), an exclusive licence and/or a non-exclusive licence for contributions from authors who are: i) UK Crown employees; ii) where BMJ has agreed a CC-BY licence shall apply, and/or iii) in accordance with the terms applicable for US Federal Government officers or employees acting as part of their official duties; on a worldwide, perpetual, irrevocable, royalty-free basis to BMJ Publishing Group Ltd ("BMJ") its licensees and where the relevant Journal is co-owned by BMJ to the co-owners of the Journal, to publish the Work in this journal and any other BMJ products and to exploit all rights, as set out in our licence.

The Submitting Author accepts and understands that any supply made under these terms is made by BMJ to the Submitting Author unless you are acting as an employee on behalf of your employer or a postgraduate student of an affiliated institution which is paying any applicable article publishing charge ("APC") for Open Access articles. Where the Submitting Author wishes to make the Work available on an Open Access basis (and intends to pay the relevant APC), the terms of reuse of such Open Access shall be governed by a Creative Commons licence – details of these licences and which Creative Commons licence will apply to this Work are set out in our licence referred to above.

Other than as permitted in any relevant BMJ Author's Self Archiving Policies, I confirm this Work has not been accepted for publication elsewhere, is not being considered for publication elsewhere and does not duplicate material already published. I confirm all authors consent to publication of this Work and authorise the granting of this licence.

For peer review only

The relationship of CT densitometry to Lung Physiological parameters and Health Status in Alpha-1 Antitrypsin deficiency; Initial report of a centralised data base of the NIHR Rare diseases Translational Research Collaborative.

Diana Crossley[1], James Stockley[2], Charlotte E Bolton[3], Nicholas S Hopkinson[4], Ravi Mahadeva[5], Michael Steiner[6], Tom Wilkinson[7], John R Hurst[8] ,Bibek Gooptu[6,9] and Robert A Stockley[2].

[1]University of Birmingham, College of Medical and Dental Sciences, Institute of Inflammation and Ageing, Centre for Translational Inflammation Research, Queen Elizabeth Hospital, Mindelsohn Way, Birmingham, B15 2WB, United Kingdom.

[2]University Hospitals Birmingham NHS Foundation Trust, Queen Elizabeth Hospital Birmingham, Mindelsohn Way, Edgbaston, Birmingham, B15 2GW, United Kingdom.

[3]Department of Respiratory Medicine, NIHR Nottingham BRC respiratory theme, School of Medicine, The University of Nottingham, City Hospital Campus, Hucknall Road, Nottingham. NG5 1PB, United Kingdom.[4]National Heart and Lung Institute, Imperial College, Royal Brompton Hospital Campus, Fulham Road, London, SW3 6NP, United Kingdom.

[5]Department of Medicine, Cambridge NIHR BRC, University of Cambridge, Cambridge, CB2 0QQ, United Kingdom.

[6]Institute for Lung Health, NIHR Leicester Biomedical Research Centre – Respiratory, University of Leicester, Glenfield Hospital, Leicester, United Kingdom.

[7] Respiratory BRU, University of Southampton, University Road, Southampton, SO17 1BJ, United Kingdom.

[8] Respiratory, Royal Free Campus, London, NW3 2PF.

[9] King's College London, Guy's Hospital Site, Great Maze Pond, London, SE1 9RT.

Corresponding Author: Professor Robert A Stockley, University Hospitals Birmingham NHS Foundation Trust, Queen Elizabeth Hospital Birmingham, Mindelsohn Way, Edgbaston, Birmingham, B15 2GW, United Kingdom. E-mail: r.a.stockley@bham.ac.uk; Tel: +44 121 371 6808. ORCID 0000-0003-3726-1207

For peer review only - http://bmjopen.bmj.com/site/about/guidelines.xhtml

Abstract (word count 299)

Objectives

To establish a data base network for the study of alpha-1 antitrypsin deficiency (AATD) and compare the results to computed tomographic (CT) lung density as the most direct measure of emphysema.

Design

A central electronic data base was established to permit the upload of anonymised patient data from remote sites. Prospectively collected CT data was recorded onto disc, anonymised, analysed at the coordinating centre and compared with the clinical features of the disease.

Setting

Tertiary referral centres with expertise in the management of AATD focused on academic Biomedical Research Units and Wellcome Clinical Research Facilities.

Participants.

Data was collected from 187 patients over 1 year from 8 UK academic sites. This included patient demographics, post bronchodilator physiology, health status and computed tomography. Analysis was undertaken at the coordinating centre in Birmingham.

Results.

Patient recruitment in the 12 months reached 94% of target (set at 200) covering the whole spectrum of the disease from those with normal lung function to very severe chronic obstructive lung disease. CT scan suitable for analysis was available from 147 (79%) of the patients. CT density, analysed as the threshold for the lowest 15% of lung voxels, showed statistically significant relationships with the objective physiological parameters of lung function as determined by spirometric GOLD severity staging ($p < 0.001$) and carbon monoxide gas transfer ($p<0.01$). Density also correlated with subjective measures of quality of life ($p=0.02$).

Conclusions.

Establishment of the network for data collection and its transfer was highly successful facilitating future collaboration for the study of this rare disease and its management. CT densitometry correlated well with the objective clinical features of the disease supporting its role as the specific marker of the associated emphysema and its severity. Correlations with subjective measures of health, however, were generally weak indicating other factors play a role.

For peer review only - http://bmjopen.bmj.com/site/about/guidelines.xhtml

Strengths and limitations of this study

The strengths of the project has been the design and delivery of a centralised registry for alpha-1 antitrypsin deficiency enabling the whole spectrum of the disease to be characterised, especially including those with more severe disease who are less able to travel long distances to single National centres of excellence.

Recruitment and data collection was rapid and 94% of target.

The study provided further validation of the specificity of quantitative analysis of lung density for the assessment of emphysema in this "rare" disease.

The limitations of the study included the slight shortfall in patient recruitment over the 1 year target and slightly incomplete post bronchodilator physiological data entry.

A reduced number of CT scans were analysed because of recent routine scans performed under different parameters. Although all patients filled in one of the health status tools this was not 100% for both.

Key Words.
Alpha-1antitrypsin deficiency, Emphysema, Computed Tomography lung physiology, Health status

INTRODUCTION

Alpha one antitrypsin deficiency (AATD) affects around 1 in 2500 individuals in the UK and increases the susceptibility to develop chronic obstructive pulmonary disease (COPD) with an early onset emphysema dominant phenotype and adult onset liver cirrhosis. AAT is the main plasma serine proteinase inhibitor and is predominantly made and secreted by the liver. It enters the tissues by simple diffusion especially in the presence of inflammation where it regulates the local activity of neutrophil proteinases (1). This is important in delicate tissue structures such as the alveolar region of the lung where

For peer review only

uncontrolled serine proteinases can destroy connective tissues leading to alveolar destruction and the development of emphysema (2).

The study and treatment of rare diseases presents many problems including patient identification, referral to centres with relevant expertise, consistent demographic characterisation and importantly the design and delivery of appropriately powered research studies and clinical trials. With this in mind the UK National Institute for Health Research commissioned a series of projects in 2014 to develop networks for the study and deep phenotyping of rare diseases with the longer term aim of providing a consistent structure to facilitate collaboration between groups and with industrial partners for research and therapeutic development.

In 1996 the ADAPT programme (Antitrypsin Deficiency and Programme for Treatment) was established in Birmingham as an investigator led, industry and research grants funded project in order to study AATD and its natural history. The programme depended on referrals, mainly from secondary care centres in the UK. Patients were invited to visit Birmingham on an annual basis for assessment as part of a clinical/ research programme (LREC 3359a). However, although successfully collating data on over 1000 deficient patients, those seen may be affected by bias due to referral patterns as well as to issues of geography and transport difficulties for annual visits. The latter issues may be especially relevant for the most severely affected patients. The ADAPT cohort may therefore underestimate the true impact, especially of the severe end of the disease spectrum. In addition the evolution of a single centre impairs delivery of near patient care, clinical trials and iterative thought, which can be handicapped by the absence of other local centres of excellence more convenient for patient attendance, management and especially, recruitment/participation in research and clinical trials.

The current article describes the AATD collaborative network funded by the National Institute of Health Research to establish a patient cohort for rare diseases collated over 1 year in the UK. In particular we wished to recruit patients with a wide range of severity, with the most severe being recruited to the closest specialist centre to minimise the issue of difficulty of travel. In particular we wished to assess lung densitometry as the most direct measure of emphysema and physiological impairment and patients perception of their health status.

For peer review only - http://bmjopen.bmj.com/site/about/guidelines.xhtml

MATERIALS AND METHODS

Birmingham acted as the coordinating centre for the National Institute for Health Research Rare Diseases Translational Research Collaboration in AATD. Links were made with academic Biomedical Research Units where a specialist with expertise in clinical trials and an interest in AATD had been identified. The network had an initial target of 200 patients with AATD and the PiZZ genotype recruited from Birmingham, Nottingham, London Brompton, London UCL/Royal Free Hospital, Cambridge, Southampton and Leicester (see figure 1). The aim was to recruit 100 patients from Birmingham with mild and moderate disease and 100 patients with severe and very severe disease (as defined by the FEV_1) from the other centres such that the distance these patients had to travel was less than attending a single central facility. A standard protocol was provided to all centres based on that used in all previous publications from the core Birmingham centre and consisted of general demographic data required for, in depth, patient characterisation. (The Lung function equipment used for the study varied between centres and is summarised for each site in the Supplement). This characterisation would also provide the basis of patient identification for inclusion in subsequent clinical trials using National standard operating procedures for lung function and specific parameters for CT acquisition.

Patients were recruited sequentially from the individual centre clinics according to the spirometric criteria outlined above. All patients gave informed consent and the study was approved for all sites by the local ethics committee (West Midlands – South Birmingham Research Ethics Committee, LREC 3359).

All subjects underwent full clinical examination, were scheduled to undergo full post bronchodilator lung function testing (according to the Association of Respiratory Technology/British Thoracic Society guidelines for quality control) including spirometry gas transfer and lung volumes, determination of current health status using well established tools (St George's Respiratory Questionnaire; SGRQ and the COPD assessment tool; CAT). Where routine high resolution Computed Tomography had not been undertaken within the previous 2 years, a scan on full inspiration was taken using the following fixed criteria:- subjects were scanned by spiral multi-slice CT of the chest in the supine position within 4 hours of administration of a short-acting bronchodilator. A low radiation dose (140kvp) was used, the slice thickness was 5mm and increments of 2.5mm, and a soft reconstruction algorithm (B30f) was applied. The anonymised data was subsequently transferred to Birmingham on disc for quantitative analysis using

For peer review only - http://bmjopen.bmj.com/site/about/guidelines.xhtml

Pulmo-CMS (Medis Medical Imaging, Leiden, Netherlands courtesy of Berend Stoel). Measurements for voxel index (% Voxels less dense than -950HU) and Perc15 (density threshold of the lowest 15% of the voxels) were assessed for the whole lung such that greater Voxel index or lower Perc (PD)15 indicated a greater amount of emphysema.

Patient and Public Involvement.

The project was approved by 5 patients who completed a Traffic Light System (TLS) research proposal review form to give their opinion on the research project and the significance of its impact on clinical practice. A further patient representative was a formal member of the steering committee. The information will be part of future presentation to the patient group and summarised for dissemination in their annual newsletter once accepted for publication. Results will also be presented at international specialty meetings. This project represented a first step towards achieving the long term aims expressed by patients as part of the European lung Foundation survey of alpha-1 antitrypsin deficiency.

Statistical Analysis

Baseline characteristics are presented as median and interquartile range (IQR). The Pearson's or Spearman rank correlation coefficient between CT density and Lung function was calculated. Analysis of variance (ANOVA) or Kruskall-Wallis was used to assess differences between grouped data. Normality of the data was tested using the Shapiro Wilk test, and the level of significance was conventionally determined at $p<0.05$.

RESULTS

Baseline characteristics

There were 187 patients recruited to the study across the eight sites within the 1 year recruitment time. Of these, 84 with severe and very severe COPD were recruited from the satellite sites and 94 mild and moderate disease (as well as 9 local patients with very severe disease) were recruited by Birmingham. Confirmed post bronchodilator spirometry was available for 185 patients and carbon monoxide gas transfer (single

For peer review only - http://bmjopen.bmj.com/site/about/guidelines.xhtml

breath) for 181. Quantitative CT scan was obtained for 147 patients and all patients completed quality of life metrics (SGRQ and/or CAT) although only 158 patients completed both.

The baseline characteristics are summarised in table 1. Data are presented as median and interquartile range to characterise the spread of patients studied. The median age was 60 years and the median post bronchodilator FEV_1 for the cohort was 51.1% predicted for age, sex, height and ethnicity (3). Of these 44 (24%) patients, (recruited from the peripheral sites) were graded as GOLD stage 4 at the time of study (4). 9 patients with AATD (5%) had an $FEV_1/FVC > 0.7$. The median CAT score for the whole group was 18.9 (12.0-25.0) and the median total SGRQ score was 45.2 (33.3-62.1).

Smoking history information was available for 178 patients. 79 were lifelong never smokers but all patients with a smoking history had ceased for more than 12 months at the time of recruitment. The median smoking history for the ex-smokers (n=99) was 17 pack years (IQR 8-27). None of the patients were receiving AAT augmentation therapy as it remains unavailable for management of Emphysema in the UK.

Cross sectional correlations

Lung function and CT densitometry

The relationship between CT density and lung function is shown in figure 2. There were significant correlations between both lung density parameters and the physiological parameters shown ($p<0.001$ all comparisons). Significant differences were also seen for CT density measurements as both the voxel index (which quantifies the proportion of low density voxels below -950 HU) and PD15 (which quantifies the threshold below which 15% of the overall voxels are sited), between each of the GOLD severity stages ($p<0.001$) (figure 3). There was no correlation between RV/TLC and any of the densitometry parameters.

Table 1. Baseline Characteristics Table. Data is shown as median and interquartile ranges together with the number of patients where data was collected. All subjects were either never smokers or had ceased at least 1 year before the time of assessment. CAT score is only given for those where the total SGRQ score was also available.

For peer review only - http://bmjopen.bmj.com/site/about/guidelines.xhtml

Clinical Variable	Number of subjects	Median (IQR)
Age (yrs)	187	60.1 (50.9-66.9)
Sex	86M 101F (46%M, 54% F)	
Pack years for ex-smokers	99	17 (8-27)
FEV_1(L)	185	1.4 (0.9-1.9)
FEV_1percent predicted	185	51.1 (30.5-67.5)
FVC(L)	185	3.6 (2.9-4.8)
FVC percent predicted	185	105.7 (88.2-123.4)
FEV_1/FVC (%)	185	36.9 (28.5-49.8)
Kco percent predicted	181	56.3 (46.0 - 69.6)
RV/TLC (%)	140	41.5 (33.0-52.7)
Voxel index -950 HU (%)	147	24.5 (15.4-35.2)
Perc15 (HU)	147	-965.2 (-974.8—950.7)
Total CAT score	158	18.9 (12.0-25.0)
Total SGRQ score	158	45.2 (33.3-62.1)

Following adjustment of all these relationships for age, sex and pack years, the relationship between density as measured by PD15 and voxel index remained significant (p<0.001) with no evidence of collinearity (see table 2).

Table 2. Multivariate linear regression models between CT density and Lung function.

Data is shown for the regression analysis of both CT parameters and baseline lung function. “pp” is the percent predicted

CT density	Lung function parameter	Unstandardised co-efficient	Standard error
PD15	FEV_1pp	0.63	0.1 (p<0.001)
	FEV_1/FVC (%)	0.94	0.1 (p<0.001)
	Kcopp	0.55	0.1(p<0.001)
Voxel index (%)	FEV_1pp	-0.48	0.1 (p<0.001)
	FEV_1/FVC (%)	-0.67	0.1 (p<0.001)
	Kcopp	-0.5	0.8 (p<0.001)

For peer review only - http://bmjopen.bmj.com/site/about/guidelines.xhtml

Quality of life measures and Lung Function

Within the cohort, the CAT test scores ranged between 1 and 39/40 (median=18.9; IQR 12-25) and related to disease severity as defined by the GOLD stage and summarised in figure4 ($p<0.0001$). The median SGRQ was 45.2 (33.3-62.1) and also related to the GOLD stage ($p<0.001$) shown in figure 4. ($p<0.001$). The median and IQR for CAT and SGRQ in each GOLD stage is summarised in table 3.

Table 3. Quality of Life data across each GOLD stage (4). The number of patients for each GOLD stage is shown together with the median and IQR data for the CAT and SGRQ total score.

GOLD Stage	Patient number	CAT median (IQR)	Patient number	SGRQ median (IQR)
FEV1/FVC>0.7	9	12 (6-21)	9	27.1 (9.7-48.0)
1	13	15 (9-21)	12	33.4 (19.3-35.7)
2	63	16 (12-21)	63	41.5 (30.9-55.4)
3	39	19 (11-25)	39	48.8 (36.5-67.3)
4	53	20 (13.5-27)	33	62.0 (36.5-67.3)

There were significant correlations (Supplemental Table 1) between both QoL measures and spirometry, as measured by FEV_1 (percent predicted), FVC (percent predicted) , FEV_1/FVC (%), and with gas transfer co-efficient, Kco (percent predicted) and gas trapping as measured by RV/TLC % ($p<0.01$ all comparisons). Supplemental figures 1 and 2 summarise the correlations for SGRQ and CAT with FEV_1 (% predicted)

Quality of life measures and CT density

Both Quality of life measures were worse the greater the emphysema as quantified by the lung density. There was a significant relationship between voxel index and CAT ($r= 0.18$, $p=0.019$) and a weaker relationship with PD15 ($r=0.15$, $p=0.043$) (figure 5). Total SGRQ correlated significantly with both measures of CT density, although again the relationship was weak ($r^2<0.1$) (see figure 5).

Data availability

No additional data is available.

For peer review only - http://bmjopen.bmj.com/site/about/guidelines.xhtml

For peer review only

Discussion

The current paper reports demographic data collated from a unique NIHR funded project to establish collaborative cohorts of patients with rare diseases focused on academic Biomedical Research Units and Wellcome Clinical Research Facilities. The main purpose of this project was to recruit 200 patients (none of whom were receiving augmentation therapy) with AATD (PiZZ genotype) to a central data base over a 1 year period as a basis for ongoing collaborative research. The aim of the collaborative was to recruit and characterise patients with AATD in depth across centres with a wide spectrum of severity. Importantly we wished to include those with severe and very severe disease (GOLD stage 3 and 4) as quantified by baseline FEV_1 (4) in the satellite centres local to their dwelling, who are more likely to refrain from travel to a more distant central facility. Of note the data for the very severe patients produced similar values and demographic relationships to those seen historically in the single centre ADAPT programme in Birmingham.

We have confirmed the feasibility of this approach with recruitment from multiple academic centres in this limited time period reaching 94% of the target. Of these 98% had confirmed post bronchodilator spirometry (in 2 it had not been documented) , 92% were able to complete gas transfer measurements and 85% had both the SGRQ and CAT measurement of health status although most patients (95%) had CAT recorded. CT scans were a unique feature of the UK patients and were performed prospectively at baseline using a fixed protocol and copied anonymously on to disc for analysis in Birmingham. This resulted in a total of 147 scans available and suitable for analysis (79% of patients) enabling the relationships to clinical parameters to be explored.

The patients showed a wide spectrum of disease including 53 patients local to the participating centres classified as GOLD stage 4. Health status showed a relationship to spirometric severity with worsening for each stage although (as expected) with a wide range and major group overlap as reported from the larger ADAPT data base (5) and other studies of non AATD COPD (6). Importantly the patients in GOLD stage 4 had a median total SGRQ score 62.0 (IQR 36.5-67.3) which was similar to that for 51 stage 4 patients recruited previously to the ADAPT data base (extracted from ref 5) with a median value of 59.1 (IQR 51.8-72.0). This suggests that our previous data was not

For peer review only - http://bmjopen.bmj.com/site/about/guidelines.xhtml

influenced by acquisition bias as a result of travel to a single centre being influenced by distance and/or patient health. In addition it indicates that GOLD 4 patients can be readily recruited from multiple sites for research, audit and appropriate clinical trials purposes.

Relationship of lung densitometry (the most direct measure of pathological emphysema) to objective parameters of lung function was similar to that seen in other studies (7,8) but better for gas transfer than spirometry (consistent with the former being a more direct measure of alveolar dysfunction as seen in emphysema) and certainly better than the weak relationship to air trapping (RV/TLC) and the subjective health status measures. COPD, even in AATD, is a multifaceted condition although classically characterised in AATD by the presence of panacinar emphysema localised to the lung bases as the dominant pathological phenotype. Lung densitometry is the most direct measure of this process and its severity as demonstrated pathologically (9). All other measures are indirect surrogate markers of emphysema and although densitometry in AATD relates to exercise capacity (10) spirometry and gas transfer (11) and health status (12) it remains the best independent predictor of mortality in this disease (13). In addition it is the most sensitive measure of emphysema progression in AATD (14) and hence its support as the primary outcome in clinical trials enabling such studies to be sufficiently powered for such a rare disease. CT has not been routinely undertaken as part of management of AATD patients largely on the basis of cost and radiation exposure. However, the radiation exposure protocol for densitometry is less than that for high resolution scans and is specific for the pathological phenotype of the COPD in AATD (namely the emphysema). Indeed the recent European Respiratory Society strategy document on AATD has confirmed it as part of the assessment for all such patients and that referral to centres with a major interest in AATD should be included in patient management (15) The relationships of CT densitometry to more recognised parameters of COPD severity and mortality are no worse and certainly better (specifically mortality) than some of the more conventional markers of the disease although still only weakly related to the subjective interpretation of the patients health.

The study was successful in enabling collaboration between academic centres by providing recruitment and in depth phenotyping of a group of patients with AATD and a wide range of physiological impairment to an agreed standard. All subjects also had

For peer review only - http://bmjopen.bmj.com/site/about/guidelines.xhtml

plasma taken and stored for the development and validation of assays to monitor serine proteinase footprints (16) and other disease specific biomarkers. In addition the network provides baseline cohort data for long term follow up and importantly a well characterised patient data base for collaboration with industry (one of the key aims for the NIHR programme). Furthermore, it conforms to the key elements outlined by the European Respiratory Society strategy document for the management of AATD (15); namely establishment of registries, comprehensive assessment and review by specialists with an interest in AATD. It also provides the background for linking with the European Union rare diseases collaborative and the European Alpha-1 Research Collaboration (EARCO) (17).

Nevertheless the study has some limitations. Several of the patients had undergone CT scanning as part of their routine clinical assessment prior to the establishment of the collaborative and hence it was not deemed ethical to repeat the procedure in the short term for densitometry analysis as part of a phenotyping study. However the parameters are set for all future scans in this patient population both at baseline and for monitoring progression. The documentation of health status was not perfectly consistent although either the SGRQ or CAT was performed in all subjects. Only 85% had the SGRQ documented whereas 95% had the CAT documented which may reflect the latter's ease of administration and practical clinical utility (4). Our analysis reported here only included those patients in whom both had been measured. Currently only the SGRQ has been accepted as a validated tool for patient reported outcomes in clinical trials (18), whereas the CAT has become an accepted measure in patient management as supported by GOLD(4) and is often also reported in clinical studies and trials. However, such measures of health status are best used to indicate a noticeable change from baseline for therapies that provide an initial and detectable impact such as bronchodilators and not therapies that modify slow progression of chronic diseases such as COPD and emphysema (5).Therefore although densitometry relates to both FEV_1 and health status (though poorly for the latter) evidence of progression and response to treatment in AATD should currently depend on CT scans as the most direct and sensitive marker of emphysema and its progression as confirmed recently in a placebo controlled trial of the benefit of augmentation therapy (19)..

For peer review only - http://bmjopen.bmj.com/site/about/guidelines.xhtml

In summary the project successfully recruited to 94% of target in the one year time frame and the majority underwent deep phenotyping to a set standard for future patient care and assessment consistent with the view of the European Respiratory Society strategy document for AATD (15). The collaboration and expertise of participants provides a firm structure for future management of AATD in the UK. Importantly it provides validation that previous patient assessment as part of the ADAPT programme reflects patients across the spectrum of disease severity and not a patient acquisition bias related to distance or travel. Furthermore, it demonstrates an intercalated network that can harmonise activity and research outcomes using standard methodologies. These points give reassurance that patient monitoring and care can be provided at multiple sites to the same standard, factors that are both critical in patient convenience and reassurance, as expressed by patients in the recent ERS AATD strategy document (15). Importantly, it provides validation of CT densitometry for characterisation of the pathological nature and severity of the disease and places this in perspective with other measures routinely used for monitoring. Because of its specificity it should become an essential part of AATD assessment (15). Finally the collaborative provides an essential, ready made resource for the delivery of research, clinical studies and trials especially for the pharmaceutical industry and a UK resource for patient/specialist research collaboration.

Author Contributions

The project concept was developed by RAS. JAS, CEB, NSH, JRH, BG, RM, MS, TAW and RAS established the network as part of the NIHR rare diseases call, collated clinical, physiological and radiological data across each contributing organisation. DC analysed the quantitative CT scans and the related data. DC drafted and refined the manuscript with RAS. All authors reviewed, modified and approved the final manuscript.

Competing Interests

None of the authors have financial relationships with any organisations in the previous three years that might have an interest in the submitted work; no other relationships or activities that could appear to have influenced the submitted work. However, RAS sits on several advisory boards for pharmaceutical companies with/ or developing, treatments for AATD and NSH is currently recruiting to a phase 2 study in these patients sponsored by Mereo Biopharma.

Funding

The Research and Network was funded by The NIHR rare diseases TRC and coordinated from the National Institute for Health Research (NIHR)/Wellcome Trust Birmingham Clinical Research Facility,. NIHR Nottingham BRC respiratory theme , Cambridge NIHR BRC ,NIHR Leicester BRC – respiratory theme, Respiratory BRU Southampton, NIHR Respiratory BRU at Royal Brompton and Harefield NHS Foundation Trust and Imperial College, London

The views expressed are those of the authors(s) and not necessarily those of the NHS, the NIHR or the Department of Health.

Data sharing

No additional data is available

Acknowledgements:

We would like to thank Anita Pye, the study coordinator for her assistance and support and Mr J Beggs the patient representative on the Steering committee.

References

1. Stockley RA.. The measurement of soluble proteins in lung secretions. Thorax. 1984; 39: 241-247.

2. Stockley RA. Alpha-1-antitrypsin and the pathogenesis of emphysema. Lung. 1987; 165: 61-77.

3. Quanjer PH, Tammeling GJ, Cotes JE, Pedersen OF, Peslin R, Yernault JC. Lung volumes and forced ventilatory flows. Eur Respir J. 1993;6 Suppl 16:5-40.

4. Vogelmeier CF, Criner GJ, Martinez FJ, Anzueto A, Barnes PJ, Bourbeau J, et al.A Global Strategy for the Diagnosis, Management, and Prevention of Chronic Obstructive Lung Disease 2017 Report. GOLD Executive Summary. Am J Respir Crit Care Med.

For peer review only

2017 Mar 1;195(5):557-582

5. Stockley RA, Edgar RG, Starkey S, Turner AM. Health status decline in α-1 antitrypsin deficiency: a feasible outcome for disease modifying therapies? Respir Res. 2018; Jul 20;19(1):137.

6. Tabberer M, Benson VS, Gelhorn H, Wilson H, Karlsson N, Müllerova H, et al. The COPD Biomarkers Qualification Consortium Database: Baseline Characteristics of the St George's Respiratory Questionnaire Dataset. Chronic Obstr Pulm Dis. 2017 Mar 13;4(2):112-123

7. Kim SS, Seo JB, Lee HY, et al. Chronic obstructive pulmonary disease: lobe-based visual assessment of volumetric CT by using standard images-comparison with quantitative CT and pulmonary function test in the COPDGene study. Radiology. 2013;266(2):626–635

8. Barjaktarevic I, Springmeyer S, Gonzalez X, Sirokman W, Coxson HO, Cooper CB. Diffusing capacity for carbon monoxide correlates best with tissue volume from quantitative CT scanning analysis. Chest. 2015;147(6):1485–1493.

9. Gevenois PA, de Maertelaer V De Vuyst P, Zanen J and Yernault JC.Comparison of computed density and macroscopic morphometry in pulmonary emphysema. Am J Resp Crit Care Med. 1995;152:653-657.

10. Dowson LJ, Newal Cl, Guest PJ, Hill SL and Stockley RA. Exercise capacity predicts health status in α1-antitrypsin deficiency. Am J Respir Crit Care Med.2001; 163: 936-941.

11. Parr DG, Stoel BC, Stolk J, Stockley RA. Pattern of emphysema distribution in a-1-antitrypsin deficiency influences lung function impairment.American Journal of Respiratory Critical Care Medicine. 2004; 170 (11): 1172-1178

12 Dowson LJ, Guest PJ, Hill SL, Holder RL and Stockley RA. High-resolution

For peer review only - http://bmjopen.bmj.com/site/about/guidelines.xhtml

computed tomography scanning in a1-antitrypsin deficiency: relationship to lung function and health status. Eur Respir J. 2001; 17: 1097-1104

13 Dawkins PA, Dowson LJ, Guest PJ, Stockley RA. Predictors of mortality in a1-antitrypsin deficiency.Thorax. 2003; 58: 1020-1026

14 Stolk J, Cooper BG, Stoel B, Rames A, Rutman O, Soliman S, Stockley R. methodology and quality control of study assessments.Retinoid treatment of Emphysema in Patients on the Alpha-1 International Registry. The REPAIR study: study design, Ther Adv Respir Dis. 2010 Dec;4(6):319-32.

15 Miravitlles M, Dirksen A, Ferrarotti I, Koblizek V, Lange P, Mahadeva M, et al. European Respiratory Society statement: diagnosis and treatment of pulmonary disease in a1-antitrypsin deficiency. Eur Respir J. 2017; 50(5):1700610

16 Newby PR, Crossley D, Stockley JA, Mumford RA, Carter RI, et al. A specific proteinase 3 activity footprint in α_1-antitrypsin deficiency. ERJ Open Res. 2019 Aug 5;5(3). pii: 00095-2019. doi: 10.1183/23120541.00095-2019

17. Miravitlles M, Chorostowska-Wynimko J, Ferrarotti I, McElvaney NG, O'Hara K, Stolk J, et al. EARCO Clinical Research Collaboration; Members of the EARCO Clinical Research Collaboration. The European Alpha-1 Research Collaboration (EARCO): a new ERS Clinical Research Collaboration to promote research in alpha-1 antitrypsin deficiency. Eur Respir J. 2019 Feb 14;53(2).

18. Food and Drug Administration. Chronic obstructive pulmonary disease: developing drugs for treatment; draft guidance for industry. Fed Regist. 2016;81(98):31946.

19.Chapman KR, Burdon JG, Piitulainen E, Sandhaus RA, Seersholm N, Stocks JM, Stoel BC, Huang L, Yao Z, Edelman JM, McElvaney NG, Group RTS. Intravenous augmentation treatment and lung density in severe alpha1 antitrypsin deficiency (RAPID): a randomised, double-blind, placebo controlled trial. Lancet 2015; 386: 360-368.

For peer review only - http://bmjopen.bmj.com/site/about/guidelines.xhtml

Figure legends

Figure 1. The NIHR rare diseases AATD network
Individual centres are shown together with their principle investigators and link to the coordinating centre in Birmingham

Figure 2. Scatter plots of the relation between Lung function and CT densitometry analysis.
Each point represents data from a single patient. The correlation coefficient (Rho) is given for each analysis using the 2 best recognised parameters for emphysema on CT scan.

Figure 3. Box plots of CT parameters by GOLD stage.
Data is shown as box plot of IQR with median value indicated by the solid line, whiskers indicate 95% data range and outliers are indicated by open circles for each GOLD stage of severity.

Figure 4. QoL parameters determined by GOLD stage.
Data is shown as box plot of IQR with horizontal median line, whiskers indicate the 95% data range and outliers are shown as open circles. Patients with no airflow obstruction are shown as a separate group

Figure 5. Scatter plots to show the relation between QoL and CT analysis. Data is shown for individual patient CAT and total SGRQ scores related to emphysema parameters. The significance of the relationships is indicated (p)

For peer review only

For peer review only - http://bmjopen.bmj.com/site/about/guidelines.xhtml

Figure 1. The NIHR rare diseases AATD network
Individual centres are shown together with their principle investigators and link to the coordinating centre in Birmingham

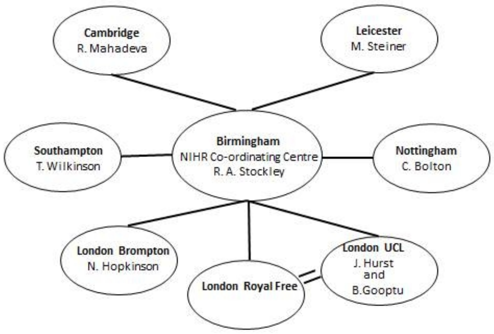

254x190mm (96 x 96 DPI)

Figure 2. Scatter plots of the relation between Lung function and CT densitometry analysis.
Each point represents data from a single patient. The correlation coefficient (Rho) is given for each analysis using the 2 best recognised parameters for emphysema on CT scan.

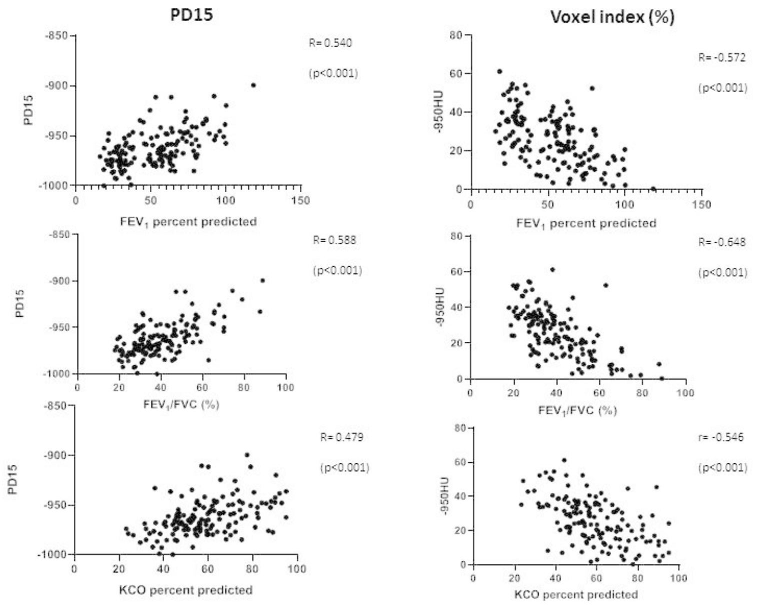

254x190mm (96 x 96 DPI)

Figure 3. Box plots of CT parameters by GOLD stage.
Data is shown as box plot of IQR with median value indicated by the solid line, whiskers indicate 95% data range and outliers are indicated by open circles for each GOLD stage of severity.

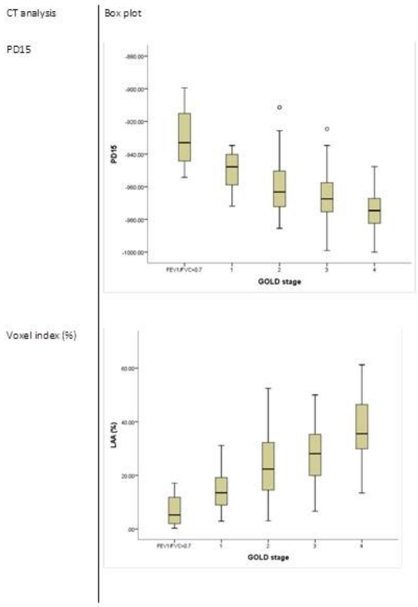

254x190mm (96 x 96 DPI)

Figure 4. QoL parameters determined by GOLD stage.
Data is shown as box plot of IQR with horizontal median line, whiskers indicate the 95% data range and outliers are shown as open circles. Patients with no airflow obstruction are shown as a separate group

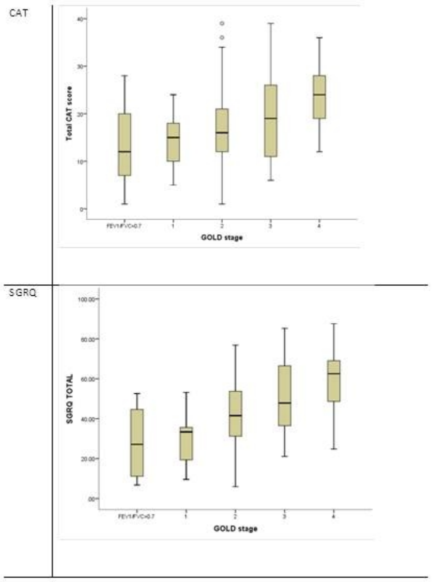

254x190mm (96 x 96 DPI)

Figure 5. Scatter plots to show the relation between QoL and CT analysis. Data is shown for individual patient CAT and total SGRQ scores related to emphysema parameters. The significance of the relationships is indicated (p)

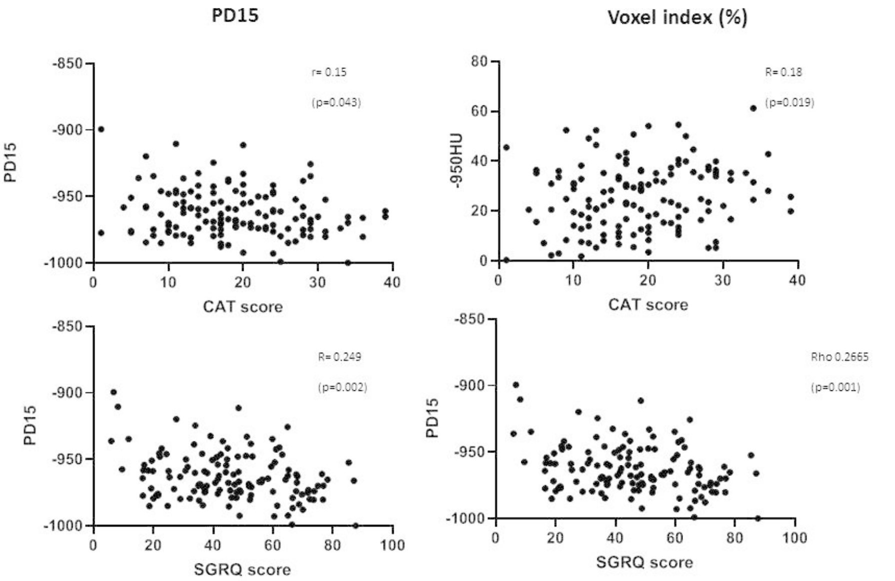

254x190mm (96 x 96 DPI)

Supplementary table and figures

The relationship between CT densitometry and clinical measures in Patients with Alpha one Antitrypsin Deficiency: the NIHR Rare diseases Translational Research Collaboration.

Diana Crossley[1], James Stockley[2], Charlotte E Bolton[3], Nicholas S Hopkinson[4], Ravi Mahadeva[5], Michael Steiner[6], Tom Wilkinson[7], John R Hurst[8] ,Bibek Gooptu[6,9] and Robert A Stockley[2].

For peer review only

Supplementary table. Summary of correlation co-efficients between Quality of Life (QoL) measures and lung function parameters.

	**CAT**	**SGRQ**
**FEV_1** (L)	-0.41*	-0.51*
**FEV_1** (%predicted)	-0.41*	-0.55*
**FVC** (L)	-0.27*	-0.32*
**FEV_1/FVC** (%)	-0.26*	-0.38*
**Kco** (mmol/min/.kPa/.L)	-0.12	-0.11
**Kco** (% predicted)	-0.26*	-0.23*
**RV** (L)	0.35*	0.46*
**TLC** (L)	-0.07	0.02
**RV/TLC** (%)	0.30*	0.32*

(*=p<0.01)

Supplemental Figure S1. Scatter plots to show the relation between SGRQ and FEV_1 percent predicted. Data is shown for individual patient total SGRQ scores related to their FEV_1.

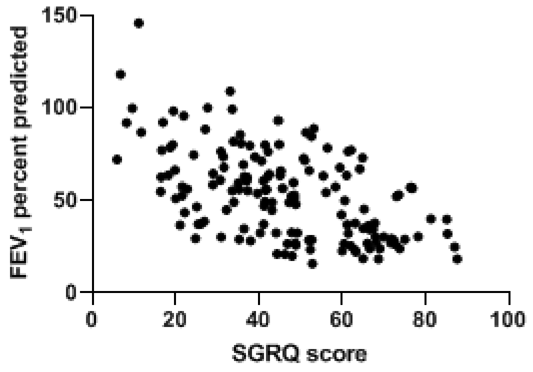

Supplemental figure S2. Scatter plots to show the relation between CAT and FEV_1 percent predicted. Data is shown for individual patient CAT scores related to their FEV_1.

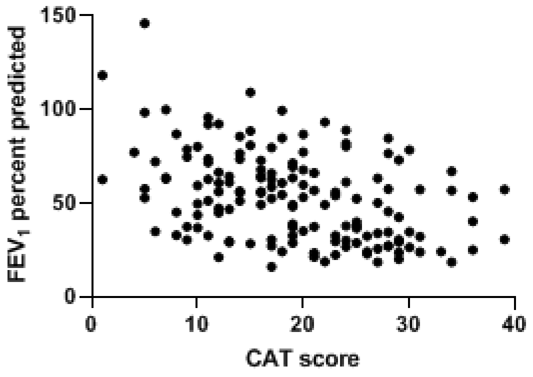

Lung Physiological equipment

Royal Free
integrated MasterScreen Pulmonary Function Testing machines (Carefusion, Germany) with the computer software JLab Lab Manager V5.32.0.
Cambridge
Jaeger Masterscreen
Royal Brompton
A CompactLab System (Jaeger, Hoechberg, Germany)
Nottingham
Spirometry - Spirometer NDD Easy On-PC
Plethysmography, DLCO - Jaeger Masterscreen PFT
Southampton
npire HDpft 4000 CAREstream Medical
Birmingham
Jaeger Masterscreen Pro lung function system (Jaeger Ltd, Hochberg, Germany)
Leicester
Medisoft HypeAir

For peer review only

Strobe check list

1. Title	Page 1
Abstract	Page 2
**Introduction**	
2. Background	Page 3,4
3. Objectives	Page 4
**Methods**	
4. Study design	Page 5,6
5. Setting	Page 5
6. Participants	Page 5
7. Variables	Page 5
8. Data sources and measurement	Page 5,6
9. Potential bias	Page 5
10. Study size	Page 5
11. Quantitative variables	Page 5
12. Statistics	Page 6
**Results**	
13. Participants	Page 6,7,8,9
14. Descriptive data	Page 8,9
15. Outcome data	Page 8,9
16. Main results	Page 9
17. Other analysis, subgroups	Page 9
**Discussion**	Pages 10,11,12
18. Summarise key findings	Pages 2,10,11
19. limitations	Pages 3,12
20. Interpretation	Pages 2,10,11,12
21. Generalisability	Page 13
22. Funding	Page 14

For peer review only
